# Structural Basis for the Diminished Ligand Binding and Catalytic Ability of Human Fetal-Specific CYP3A7

**DOI:** 10.3390/ijms22115831

**Published:** 2021-05-29

**Authors:** Irina F. Sevrioukova

**Affiliations:** Department of Molecular Biology and Biochemistry, University of California, Irvine, CA 92697-3900, USA; sevrioui@uci.edu

**Keywords:** cytochrome P450, CYP3A7, surface mutant, crystal structure, protein conformation, protein plasticity

## Abstract

Cytochrome P450 3A7 (CYP3A7) is a fetal/neonatal liver enzyme that participates in estriol synthesis, clearance of all-trans retinoic acid, and xenobiotic metabolism. Compared to the closely related major drug-metabolizing enzyme in adult liver, CYP3A4, the ligand binding and catalytic capacity of CYP3A7 are substantially reduced. To better understand the structural basis for these functional differences, the 2.15 Å crystal structure of CYP3A7 has been solved. Comparative analysis of CYP3A enzymes shows that decreased structural plasticity rather than the active site microenvironment defines the ligand binding ability of CYP3A7. In particular, a rotameric switch in the gatekeeping amino acid F304 triggers local and long-range rearrangements that transmit to the F-G fragment and alter its interactions with the I-E-D-helical core, resulting in a more rigid structure. Elongation of the β_3_-β_4_ strands, H-bond linkage in the substrate channel, and steric constraints in the C-terminal loop further increase the active site rigidity and limit conformational ensemble. Collectively, these structural distinctions lower protein plasticity and change the heme environment, which, in turn, could impede the spin-state transition essential for optimal reactivity and oxidation of substrates.

## 1. Introduction

The cytochrome P450 (CYP) 3A subfamily accounts for ~30% of the total CYP content in adult liver and plays a major role in drug metabolism [1]. This subfamily includes four enzymes: CYP3A4, CYP3A5, CYP3A7 and CYP3A43. CYP3A4 is the major liver and intestinal isoform that is the primary drug-metabolizing CYP. Expression of functional CYP3A5 depends on the ethnic group [2] and frequently occurs in extrahepatic tissues, where it can impact in situ drug metabolism [3]. Likewise, CYP3A43 is primarily an extrahepatic isoform, constitutively expressed at low levels [4]. In contrast, CYP3A7 is the major CYP and CYP3A isoform in fetal and neonatal liver [5,6]. Significant levels of CYP3A7 can also be found in other fetal tissues, as well as in developing infants up to 24 months post-gestational age (reviewed in [7]). The main function of CYP3A7 during fetal development is regulation of placental estriol synthesis via 16α-hydroxylation of dihydroepiandrosterone (DHEA) and its sulfate derivative (DHEAS) [8,9], as well as clearance of all-trans retinoic acid (atRA) [10], the primary ligand for embryonic nuclear retinoic acid receptors. During the first week after birth, CYP3A7 expression progressively declines, while CYP3A4 expression is simultaneously activated through transcriptional regulation [11]. Despite high sequence similarity (~93%) and overlapping substrate specificity with CYP3A4, fetal-specific CYP3A7 has a significantly lower catalytic ability and generates an altered regioselectivity profile [12]. As a result, the CYP3A7-to-CYP3A4 switch can largely impact xenobiotic metabolism and toxicity in neonates and developing infants, which is of vital importance in pediatric pharmacology [5].

To date, only a few investigations have been conducted on recombinant CYP3A7 [13,14,15,16], whose structure–function relationships remain poorly understood. The current study was undertaken to fill this knowledge gap. Since the wild type (WT) CYP3A7 resists crystallization, site-directed mutagenesis of surface residues was conducted to find a variant that promotes crystal growth. One such variant, R69G/C77G/K244E/K421A/K422A/K424A (CYP3A7mut), was produced, and its 2.15 Å dithiothreitol-bound structure was solved, providing the first insights into the molecular architecture and enabling comparative analysis of CYP3A enzymes. Based on our data and the previously reported functional results, we propose that decreased conformational plasticity and higher rigidity of the active site are the main reason for the diminished ligand binding and catalytic ability of CYP3A7.

## 2. Results and Discussion

### 2.1. Modification of Surface Residues in CYP3A7

Recombinant CYP3A7 lacking the membrane tether (residues 3-22) is well expressed in *Escherichia coli* and can be isolated in a highly purified form (A_417/280 nm_ > 1.6). However, the WT protein does not produce crystals, even in the presence of small heme-ligating molecules, such as imidazole, phenyl-imidazole, or dimethyl sulfoxide, known to stabilize and promote CYP crystallization. This is in contrast to Δ3-22 CYP3A4, which willingly crystallizes in the ligand-free and ligand-bound forms. Therefore, CYP3A4 crystal packing was analyzed to identify divergent surface residues in CYP3A7 that could potentially interfere with crystal formation. Two such residues, R69 and K244, were replaced with Gly and Glu, respectively, to mimic those in CYP3A4. Since neither single nor double substitutions promoted the crystallization of CYP3A7, another CYP3A4-mimicking mutation, C77G, was introduced. The triple mutant had higher expression levels (by ~20%) and produced non-diffracting crystalline formations under several conditions, which we were unable to optimize.

In parallel, the Surface Entropy Reduction prediction (SERp) server (http://services.mbi.ucla.edu/SER/, accessed on 16 May 2019) was used to identify clusters of high-entropy surface residues (Arg, Lys, and Glu), which, upon replacement with alanine, can promote protein crystallization [17]. Previously, this strategy has been successfully applied to CYP3A4, whose K282A/K285A and K421A/K424A surface mutants were indispensable for solving ligand-bound structures that could not be obtained with the WT protein [18,19]. In CYP3A7, the top scored high-entropy surface cluster was K421/K422/K424 (SERp score 6.58), but, again, neither single, double, or triple alanine substitution promoted crystallization. This prompted us to combine K421A/K422A/K424A and R69G/C77G/K244E mutations. The resulting sextuple mutant was well expressed in *E. coli*, stable upon purification, and produced diffracting crystals.

### 2.2. Impact of Surface Mutations on CYP3A7 Properties

**Figure 1:**
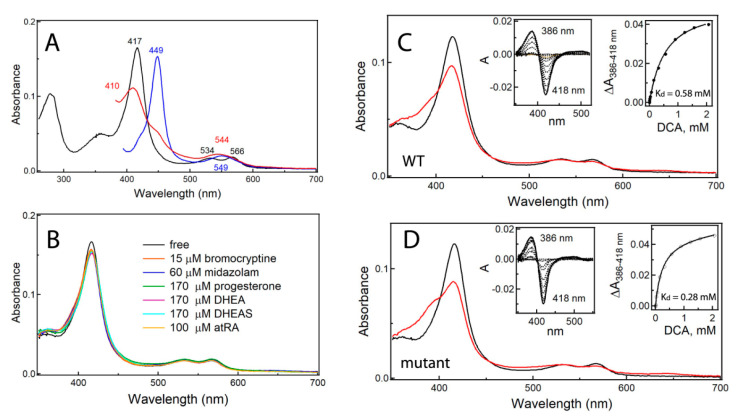
(**A**) Absorbance spectra of 1.5 μM ferric (black), ferrous (red), and ferrous-CO bound (blue) CYP3A7mut. Absorbance maxima are indicated. Spectra were measured in 0.1 mM potassium phosphate buffer pH 7.4, supplemented with 20% glycerol and 1 mM dithiothreitol, and were similar to those of WT CYP3A7. (**B**) Lack of changes in the Soret band of CYP3A7 in the presence of substrates at saturating concentrations. (**C**,**D**) Spectral changes induced by DCA in the WT and mutant CYP3A7, respectively. Black and red spectra were recorded before and after equilibrium titrations, with the final DCA concentration of 2.1 mM. Left insets are difference spectra recorded after each DCA addition. Right insets are titration plots with hyperbolic fits, from which the dissociation constants (K_d_) for DCA were derived.

To ensure that the mutant structurally and functionally resembles WT and is suitable for crystallographic studies, we investigated the mutational impact on spectral, catalytic, and molecular properties of CYP3A7. Absorbance spectra of ferric, ferrous, and ferrous CO-bound CYP3A7mut (Figure 1A) were nearly identical to those of WT. This and the high similarity of circular dichroism spectra (Supplementary Figure S1A) indicate that mutations do not affect protein folding and heme incorporation. Gel filtration experiments, in turn, showed that both WT and the mutant form dimers and higher molecular weight oligomers in solution (Supplementary Figure S1B).

An important diagnostic tool for assessing functional properties of CYPs is the type I low- to high-spin shift that accompanies the binding of substrates near the heme iron. As shown in Figure 1B, DHEA, DHEAS, atRA, and other type I ligands that induce the full type I spectral change in CYP3A4 have little effect on WT and mutant CYP3A7. As we found earlier, deoxycholate (DCA), the major secondary bile acid formed by gut microbiota, serves as a type I ligand for the full-length CYP3A7, leading to a ~30% high-spin conversion at saturating concentrations [14]. This natural substrate was used for equilibrium titrations to evaluate the ligand-binding properties of CYP3A7. As seen in Figure 1C,D, spectral changes induced by DCA in the WT and mutant protein were highly similar, and the derived dissociation constants (K_d_) were in the same range: 0.58 ± 0.3 and 0.28 ± 0.2 mM, respectively. The corresponding K_d_ for CYP3A4 was several-fold higher (1.54 mM [14]). Further, DCA binding to CYP3A4 was cooperative, but non-cooperative for CYP3A7. More notably, during association of DCA to CYP3A7, equilibrium was reached quickly after compound mixing, whereas up to 15–20 min were required for completion of the reaction involving CYP3A4. This suggests that, unlike CYP3A4, CYP3A7 does not undergo slow structural readjustments to optimize the DCA binding, possibly due to a larger/more accessible and/or less flexible active site.

To evaluate the mutational impact on the catalytic activity of CYP3A7, the rate of 7-benzyloxy-4-(trifluoromethyl)coumarin (BFC) debenzylation was measured by following formation of a fluorescent product. When cytochrome P450 reductase (CPR) was used as an electron donor, CYP3A7mut retained ~33% of the WT activity, with a <2-fold decrease in the catalytic efficiency (V_max_/K_m_; Table 1). One reason for the activity decline could be alteration of the redox partner binding site, as elimination of basic K421, K422, and K424 on the proximal face of CYP3A7 may hinder CPR association. To confirm this, the BFC activity was measured in the presence of an oxygen surrogate, cumene hydroperoxide (CuOOH), known to effectively support P450 activity [20]. This reaction proceeded 10-fold faster, with no significant difference in V_max_ and K_m_ for the WT and mutant CYP3A7 (Table 1). Still, even the CuOOH-supported reaction was an order of magnitude slower than the CPR-supported turnover of CYP3A4: 0.035 vs. 0.33 min^−1^ [21], respectively. Based on functional and spectral results, we conclude that the surface mutations have minimal, if any, CYP3A4-mimicking effect and do not significantly alter the active site and overall architecture of CYP3A7. Thus, CYP3A7mut is suitable for crystallographic studies and could serve as a reliable WT model.

### 2.3. Crystal Structure of CYP3A7

**Figure 2:**
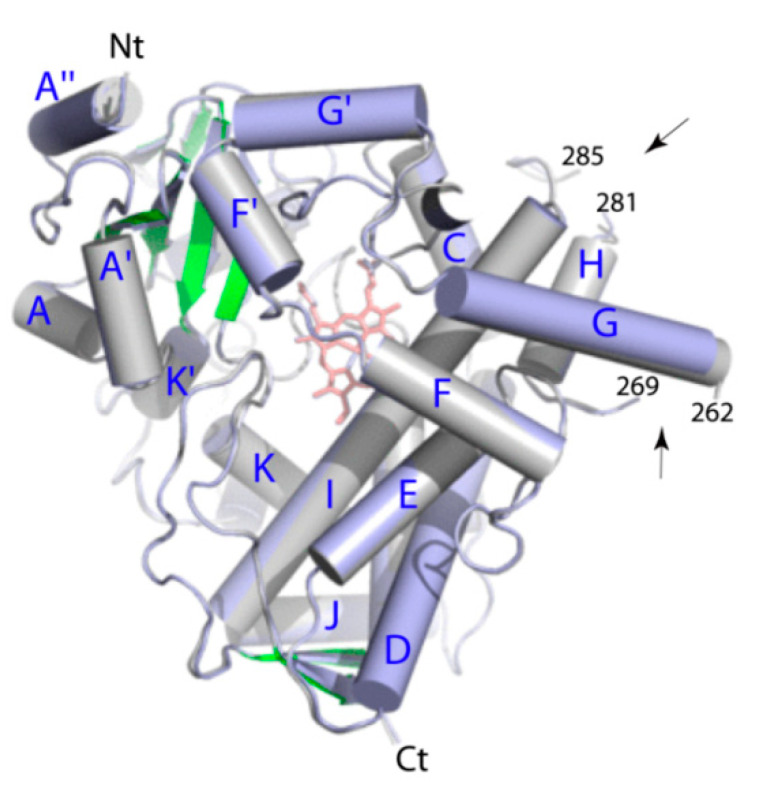
Superposition of two crystallographically independent molecules of CYP3A7. Molecules A and B and shown in gray and lavender, respectively. Helices and the N- and C-termini are labeled. The polypeptide breakage sites are indicated by arrows.

CYP3A7mut crystallized in space group C222, with two well-defined molecules per asymmetric unit. The X-ray structure was refined to 2.15 Å resolution and the *R_work_* and *R*_free_ values of 20.3 and 25.3, respectively (Table 2). When superimposed, molecules A and B were virtually identical (Figure 2), with the r.m.s. deviation between the Cα atoms of 0.636 Å. The N- and C-termini and two short internal fragments, 263–268/282–284 in molecule A and 263–267/282–283 in molecule B, were not seen due to thermal disorder. Out of six mutated residues, only three were at the crystal packing interface (Supplementary Figure S2) and allowed closer contacts with the symmetry-related molecules either due to a shorter/lacking side chain (R69G and K422A) or through partial basic charge neutralization (K244E). The C77G, K421A, and K424A residues do not form intermolecular contacts and may not directly promote crystal formation.

**Figure 3:**
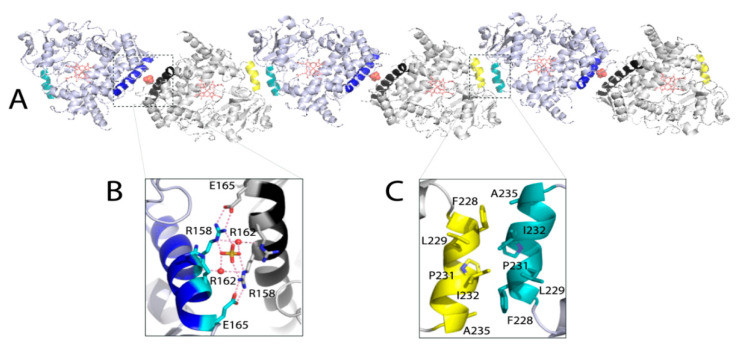
(**A**) Packing of CYP3A7 in the crystal lattice. Intermolecular contacts are mediated by the D-helices (in blue and black) and G′-helices (in yellow and teal). Sulfate ions bridging the D-helices are shown in cpk representation. (**B**,**C**) Magnified views at the polar and hydrophobic interface formed by the antiparallel D- and G′-helices, respectively. The interacting residues are displayed and labeled. Red dotted lines are H-bonds; red spheres are water molecules.

Since Δ3-22 CYP3A7 forms dimers in solution (Supplementary Figure S1B), crystal packing was analyzed to better understand how protein dimerization might occur. Two types of crystallographic dimers were identified (Figure 3). One interface is formed by the antiparallel D-helices and contains a sulfate ion, linking the opposing R158 guanidine groups through direct and water-mediated H-bonds (Figure 3B). The nearby R162 is not poised for the H-bond formation but could provide additional charge neutralization. Such an arrangement enables formation of two intermolecular R158-E165 salt bridges that further strengthen the interface. Another contact area is hydrophobic and formed by residues from the antiparallel G′-helices (Figure 3C). Because the sulfate ion was strictly required for crystallization but not dimerization of CYP3A7, it can be concluded that intermolecular contacts at the polar interface promote crystal formation, whereas hydrophobic contacts mediated by the G′-helix likely lead to CYP3A7 dimerization in solution.

**Figure 4:**
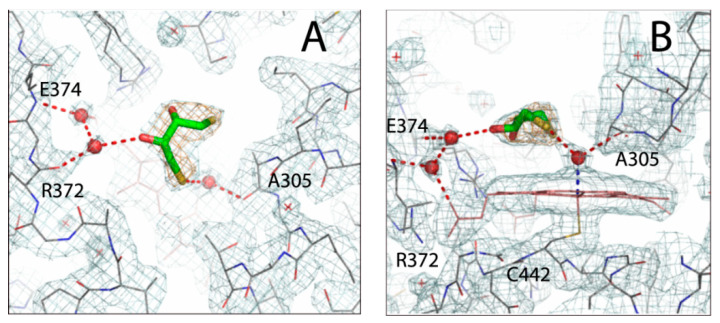
Top (**A**) and side views (**B**) at the DTT molecule bound to the active site of CYP3A7 (molecule A). DTT (in green sticks) binds above the heme and establishes water mediated H-bonds (red dotted lines) with the main chain atoms of A305, R372, and E374 and the heme iron. Red spheres and crosses are water molecules. Orange mesh around DTT is a simulated annealing omit map contoured at 3 σ level; gray–cyan mesh is 2Fo-Fc electron density map contoured at 1 σ level.

A bulk of positive electron density above the heme was seen in both molecules of CYP3A7, indicating association of a small polar ligand. Among components of purification and crystallization solutions, only dithiothreitol (DTT) could fit well into the density (Figure 4). Therefore, the DTT-bound structure was refined and presented here. The DTT ligand is held in place via water-mediated H-bonds that, on one side, link the hydroxyl group to the main chain atoms of R372 and D374 and the heme propionate and, on the opposite side, connect the thiolate sulfur to the iron and the A305 carbonyl. In molecule B, DTT is in a similar environment, but the distance between the sulfur atom and the heme-bound water ligand is longer: 2.9 Å vs. 2.6 Å in molecule A. Overall, molecule A was better defined and, therefore, it was used for structural comparison with CYP3A4 and CYP3A5.

### 2.4. Unique Features of CYP3A7

#### 2.4.1. Divergence in the F-G Fragment

**Figure 5:**
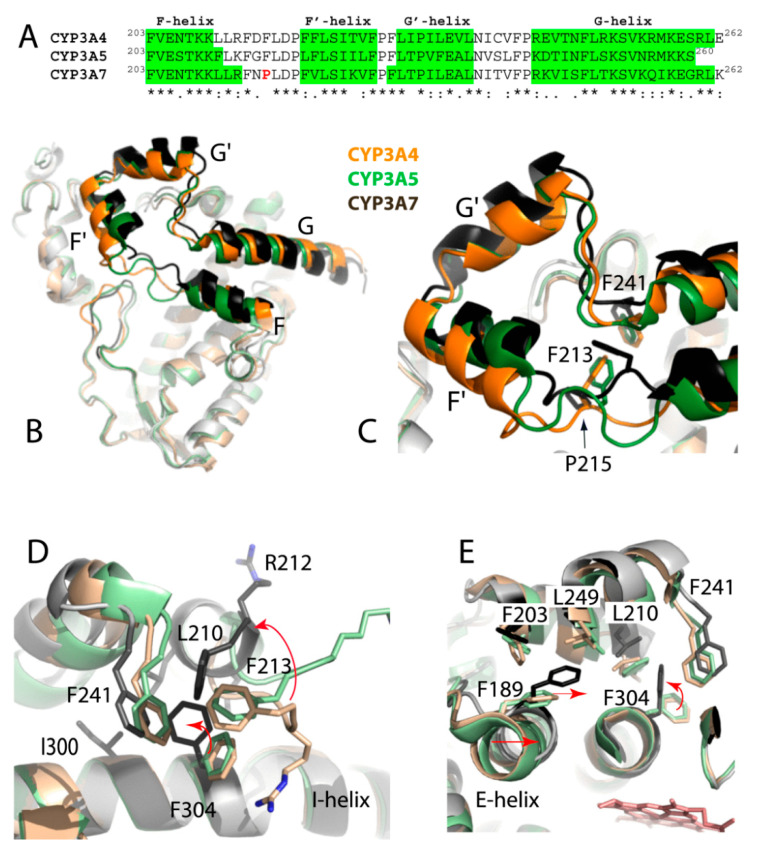
(**A**) Sequence alignment of the F-G fragments in CYP3A enzymes. (**B**) Superposition of CYP3A4 (5vcc; orange), CYP3A5 (6mjm; green), and CYP3A7 (molecule A; gray/black). (**C**) Magnified view of the distinctly folded F-F′-G′-G segments. (**D**) F304-centered cluster formation in CYP3A7 (depicted in gray/black). Arrows show rotameric change in F304 from “inward” in CYP3A4/5 to “upward” in CYP3A7, where a subsequent conformational switch in the 210–213 fragment promotes clustering of L210, L211, F213, and F241. (**E**) Side view showing that rotameric switch in F304 triggers movement of F189 and the E-helix (indicated by arrows).

CYP3A enzymes have high sequence identity (>82%) and share the same fold. When their 3D-structures are superimposed, the r.m.s. deviation between the Cα atoms is 1.36 Å for both CYP3A4/3A7 and CYP3A5/3A7 pairs. The most notable difference is in the folding of the F-F′-G′-G helix/loop region (Figure 5), serving as an upper wall of the active site. In CYP3A7, the F-F′ connector is shortened due to the F-helix extension, and its central F215, part of the phenylalanine cluster in CYP3A4 [22], is replaced with proline (Figure 5A). Relative to CYP3A4/5, there is a lateral shift in the F′- and G′-helices and connecting loops (Figure 5B,C), likely triggered by the F304-centered cluster formation (explained below). As a result, the closely positioned F-F′ and G′-G loops establish more intricate interactions, leading to partial immobilization and higher rigidity of the F-G fragment.

#### 2.4.2. Formation of the F304-Centered Hydrophobic Cluster

F304 is the active site residue located on the I-helix close to the catalytic center. In CYP3A4/5, F304 serves as a gate keeper and regulates access to the heme through rotameric changes in the side chain. In the water- or small-molecule-bound structures, the phenyl ring points toward the cofactor (“inward” rotamer) but swings away (“upward” rotamer) to allow association of bulky ligands [22,23,24,25,26]. In DTT-bound CYP3A7, F304 adopts the upward conformation, and its phenyl ring is stacked in a hydrophobic cavity formed by L210, L211, F213, and F241 (Figure 5D). Computer modeling showed that the upward-to-inward F304 rotamer change does not cause steric clashing with DTT (Supplementary Figure S3). Thus, the conformational switch in F304 is likely triggered by the distinct fold/composition of the F-G fragment rather than association of DTT. One subsequent event is the flipping of the 210–213 fragment, which leads to an outward orientation of R212 and a less restricted access to the heme. Another notable structural change occurs on the opposite side of the I-helix, where F189 moves toward F304 by >3.0 Å (Figure 5E). This prompts reorganization in the neighboring residues (F203 from the F-helix, L249 and V253 from the G-helix, M275 from the H-helix, and L295 and M296 from the I-helix) to optimize hydrophobic interactions surrounding F304.

#### 2.4.3. Tighter Packing of the I-E-D-Helix Bundle

**Figure 6:**
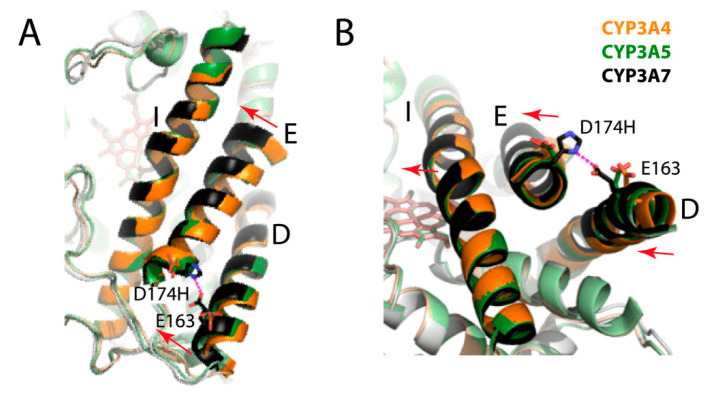
Top (**A**) and side views (**B**) on the I-E-D-helix bundle in CYP3A4 (5vcc), CYP3A5 (6mjm), and CYP3A7 (molecule A). In CYP3A7, there are helical shifts (indicated by arrows) and the newly formed H-bond between E163 and H174 (dotted line) that stabilizes the more compact and less stretchable conformation.

Because F189 is part of the E-helix, its movement toward F304 drags the E-helix toward the I-helix as well. This is an important outcome that leads to tighter packing and decreased dynamics of the I-E-D-helix bundle, hereinafter referred to as the helical core (Figure 6). The largest shift is observed for the distal parts of the E- and D-helices, which move toward the I-helix by 3.1 and 2.0 Å, respectively. In CYP3A4/5, electrostatic repulsion between acidic residues located at the proximal ends of the E- and D-helices (D174 and E163, respectively) helps to hold the helices apart from one another. In CYP3A7, D174 is replaced with histidine and H-bonds with E163 (Figure 6), which immobilizes the helical core relative to CYP3A4. Positional changes in the central part of the I-helix are smaller but still important, as they restrict access to the catalytic center. Compared to CYP3A4, the Cα atoms of I301, A305, and T309 are closer to the heme iron by 0.43, 0.73, and 0.97 Å, respectively (Supplementary Figure S4). The corresponding residues in CYP3A5 are at intermediate positions. Since the D-helix mediates crystallographic contacts (Figure 3B), its positioning could be affected to some extent by crystal packing. The I-E-D-helix bundle conformation, however, is mainly defined by the E-helix, which does not form intermolecular contacts.

**Figure 7:**
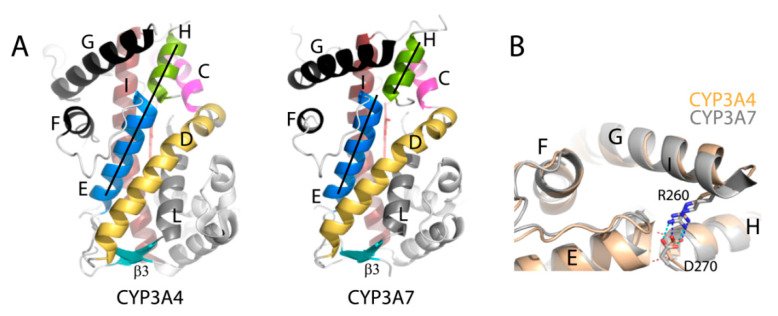
(**A**) Side views of CYP3A4 (5vcc) and CYP3A7 (molecule A) show relative positioning of the E- and H-helices. (**B**) Contact site between the F-, G-, E-, and H-helices. In CYP3A4, two hydrogen bonds are formed between the main chain atoms of the T187/F271 and A188/L272 pairs (depicted as red dotted lines), which together with the R260-D270 salt bridge could mediate communication between the helices. In CYP3A7, the E-helix shift leads to H-bond disruption/elongation and decreases the E/H-helix contact area by half.

Other important differences in the E- and H-helices are two inter-helical hydrogen bonds that are present in CYP3A4 but missing or weakened in CYP3A7: T187:O-F271:N and S188:O-L272:N (Figure 7). This weakens the E/H-helix interaction in CYP3A7 and decreases their contact area by half. Even though the R260-D270 salt bridge connecting the G- and H-helices remains in place, the F/G/E/H-interhelical communications are likely to be perturbed. Thus, the F304-centered cluster formation and subsequent structural reorganization not only rigidify/immobilize the helical core but could also alter its sensitivity and response to conformational changes in the F-G segment, the key element involved in substrate binding/recognition and allosteric interactions in CYP3A4 [27,28,29].

#### 2.4.4. Other Structural Distinctions

**Figure 8:**
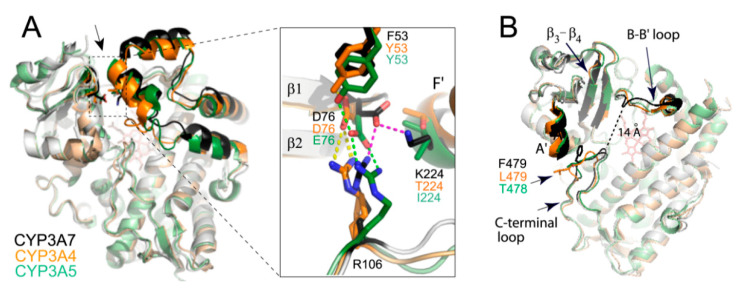
(**A**) General and magnified views at the substrate channel in CYP3A4 (5vcc; orange), CYP3A5 (6mjm; green), and CYP3A7 (molecule A; gray/black). The F-G segment and the β_1_-β_2_ connecting loop are highlighted in brighter colors. Entrance to the substrate channel is indicated by an arrow. Due to Y53F and T/I224K substitutions in CYP3A7, its D76 establishes a different H-bonding network that links the opposite channel walls. (**B**) Because of distinct folding of the B-B′ and C-terminal loops, the catalytic cavity in CYP3A7 is shaped differently and narrower than in CYP3A4/5. Elongation of the β_3_-β_4_ strands further decreases plasticity of the active site. The F-G segment was removed for clarity.

In CYP3A enzymes, the F′-helix is one of the most mobile elements that lines the wall of the substrate channel and actively participates in substrate binding. CYP3A7 has two substitutions, T/I224K in the F′-helix and Y53F in the opposing A′-helix, which change the width and H-bonding pattern in the central part of the substrate channel. In CYP3A4/5, D76 from the β_1_-β_2_ connecting loop is engaged in polar and electrostatic interactions with Y53 and R106, located on the same side of the channel (Figure 8A). CYP3A7 has nonpolar phenylalanine at position 53, owing to which D76 adopts a different rotamer to engage with R106 and K224. As a result, the mid portion of the substrate channel becomes more constricted and interlinked.

**Figure 9:**
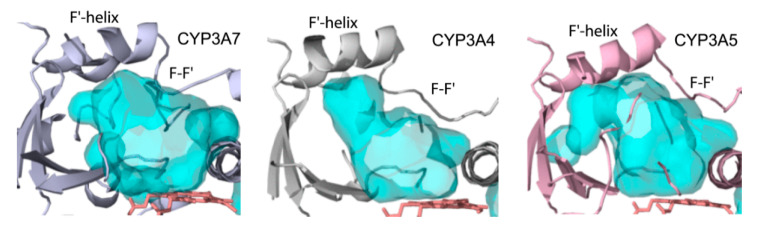
Differently shaped active site cavities in CYP3A enzymes.

Further, compared to CYP3A4/5, the β_3_ and β_4_ strands in the β-domain are elongated by 2-3 residues (Figure 8B). This strengthens H-bonding in the β1-sheet and makes the active site wall more rigid. The B-B′ loop, lining the adjacent wall, is also reshaped due to restructuring of the G′-G connecting segment. Finally, the C-terminal loop is of the same length as in CYP3A4/5 but more elongated and extends deeper into the active site due to steric constraints imposed by the I-helix on one side and the A′-helix and its bulky F479 (L479 in CYP3A4; T478 in CYP3A5) on the opposite side (Figure 8B). This double-sided squeeze rigidifies the C-terminal loop and decreases the length of the active site cavity: distance between the closest fragments of the C-terminal and B-B′ loops is ~14 vs. 17 Å in CYP3A4 and 20 Å in CYP3A5 (Figure 8B). Still, CYP3A7 has a spacious active site: 3197 vs. 2763 Å^3^ in CYP3A4 and 4089 Å^3^ in CYP3A5 (calculated with MOLE [30]). It should be noted, however, that the cavity size is largely affected by the R212 orientation (inward in CYP3A4 and outward in CYP3A5/7) and by positioning of the F-F′ loop, being at the highest level in CYP3A7 and the lowest in CYP3A4 (Figure 9). Additionally, as previously reported [31], the CYP3A5 active site extends to the surface via a solvent channel, which substantially increases the cavity size.

### 2.5. Functional Implications

Compared to CYP3A4/5, the catalytic efficiency of CYP3A7 with most substrates is significantly reduced, as evident from the substantially lower V_max_ and higher K_m_ values [12,13] (Table 1). Metabolic activity of CYP3A7 is greater only with two natural substrates, DHEAS [9,32] and atRA [10], suggesting the evolutionary role of CYP3A7 in embryonic development and protection against DHEAS/atRA-induced embryotoxicity, teratogenicity, and premature birth. However, even DHEAS, atRA, and other typical CYP3A substrates do not induce feasible high-spin transition in CYP3A7 (Figure 1B). A similar trend was observed for type II ligands that coordinate to the heme via basic nitrogen atoms, as both imidazole-containing and triazole antifungal drugs were found to bind to CYP3A7 with a considerably lower affinity relative to that for CYP3A4 and, in some cases, with no spectral evidence for association [15]. The crystal structure of CYP3A7 helps to understand why such closely related CYP3A enzymes differ so drastically in the ligand binding and catalytic ability.

**Figure 10:**
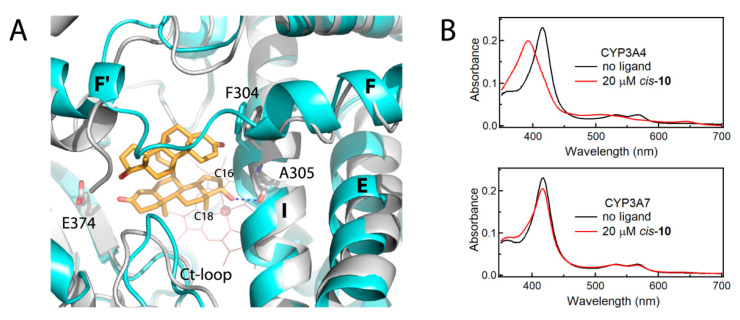
(**A**) Displacement of the I-helix in CYP3A4 bound to the high affinity type I ligand, *cis*-**10** (7lxl structure; in gray). This conformational change would be impossible in CYP3A7 (in cyan) due to tighter packing of the I-E-D-helical core. The *cis*-**10** ligand is shown in orange sticks; the blue dotted line is an H-bond linking the sterol hydroxyl group to the A305 carbonyl. Changes in the CYP3A4 helical core triggered by other ligands are demonstrated in Supplementary Figure S4. (**B**) Complete and partial (~20%) high-spin shift induced by *cis*-**10** in CYP3A4 and CYP3A7, respectively. Absorbance spectra of 2 μM proteins were measured in 0.1 mM potassium phosphate pH 7.4, supplemented with 20% glycerol and 1 mM dithiothreitol.

It is generally accepted that P450 activity is defined by both the accessibility/substrate binding capacity of the active site and protein dynamics. The importance of structural plasticity for productive ligand binding has been well demonstrated for CYP3A4. For instance, binding of the small substrate, midazolam, triggers global structural readjustments that transmit from the F-G segment to the adjacent D-, E-, H-, and I-helices and result in reshaping/collapse of the active site [33]. In contrast, association of large type II inhibitors, such as ritonavir and ketoconazole, causes the active site expansion, mainly achieved through the I-helix bending and conformational changes in the F-F′-helix/loop region [24,25]. The largest I-helix displacement, up to 2.3 Å, was observed in CYP3A4 bound to inhibitor **4c** (6dab structure) and accompanied by smaller movements in the E- and D-helices (Supplementary Figure S5). A smaller but still substantial I-helix distortion (~1.2 Å) was also observed in CYP3A4 bound to the high affinity type I ligand, testosterone dimer *cis*-**10** (K_d_ of 0.37 µM; 7lxl structure) [34]. This conformational change allowed the proximal sterol to bind closely and near parallel to the heme, establish a stabilizing H-bond with the A305 carbonyl, and place the C16 and C18 atoms of the D-ring (β-side) suitably for oxidation (3.9–4.1 Å away from the iron; Figure 10). Thus, stretchability of the I-helix and flexibility of the I-E-D-helical core might be one of the prerequisites for promiscuity and efficient/productive substrate binding.

In CYP3A7, collapse or expansion of the active site cannot be attained to the same extent as in CYP3A4. One reason is the uniquely folded F-G segment, which becomes interlinked with the helical core upon F304-triggered rearrangement (Figure 5 and Figure 6). The most important aftermath of short- and long-range repercussions is rigidification of the F-G fragment and the I-E-D-helix bundle, which could alter interhelical communications and lower protein plasticity and conformational dynamics. Extension of the β_3_-β_4_ strands, the narrower and interlinked substrate channel, and steric constraints in the deeply protruding C-terminal loop (Figure 8) are other factors that further increase rigidity of the active site and limit the conformational ensemble. Together, these global effects could modulate the heme environment and preclude high-spin conversion in the presence of substrates and other type I ligands (Figure 1B and Figure 10B). In other words, based on spectral, functional, and structural data, we suggest that the lower flexibility and inability of CYP3A7 to change the shape of the active site to optimize substrate binding and allow spin transitions is the primary reason for the diminished catalytic activity and distinct stereo/regioselectivity profiles [12,13,14,16]. One way to test this hypothesis would be determination of crystal structures of CYP3A7 bound to natural substrates with the known sites of metabolism, e.g., DHEAS, atRA, and testosterone. Although highly challenging, this approach would provide direct insights on the ligand-triggered structural changes and help better understand substrate specificity of CYP3A enzymes.

### 2.6. CYP3A7 Polymorphism

**Figure 11:**
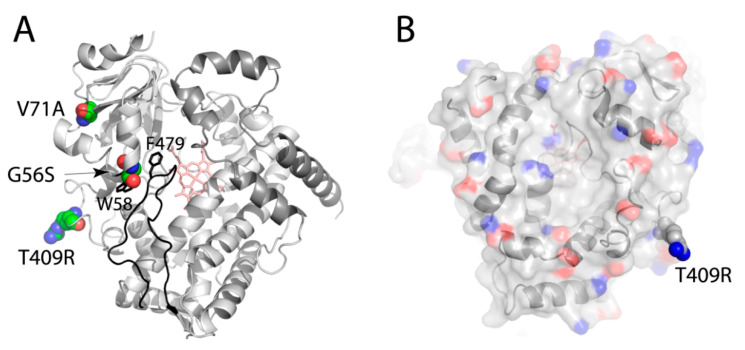
Naturally occurring CYP3A7 mutations. (**A**) Distal view showing location of mutated residues (in cpk representation). The C-terminal loop is rendered in black. Side chains of W58 and F479 are displayed to demonstrate steric clashing with S56. (**B**) Charge distribution on the proximal surface of CYP3A7. Positively and negatively charged groups are in blue and red, respectively. The T409R mutation introduces an additional positive charge, which could assist docking of the redox partner, cytochrome P450 reductase.

Interindividual variability in CYP3A7 expression occurs mostly due to mutations in the promoter region [35,36,37,38]. The most significant CYP3A7*1C allele results in functional CYP3A7 expression in adults and influences metabolism of endogenous sex hormones and outcomes in various malignancies [37]. A comprehensive survey of sequence variations in the *CYP3A7* gene identified only three single nucleotide polymorphisms in the exon regions that result in G56S, V71A, and T409R mutations [38]. The functional role of the first two mutations has not been investigated, but in silico analysis suggests that the G56S substitution might be impactful. G56 is located near W58 at the end of the A′-helix and in close contact with F479 from the C-terminal loop. Serine at position 56 would impose steric hindrance on F479 and W58 (Figure 11A), which might affect the folding of the A′-helix and C-terminal loop and alter the active site architecture. V71, on the other hand, is part of the hydrophobic patch on the β-domain surface. In silico modeling showed that the V71A substitution may somewhat change the local environment but is unlikely to have a notable structural or functional effect.

The T409R variant (CYP3A7*2 allele) is more common, and its frequency ranges from 8% in white to 62% in Tanzanian individuals [39]. Moreover, the CYP3A7*2 allele is in linkage disequilibrium with CYP3A5*1 and results in a haplotype characterized by CYP3A5 expression and increased enzymatic activity of CYP3A7. T409 is a surface residue whose replacement with arginine changes the electrostatic potential because of an additional positive charge (Figure 11B). The proximal face serves as a docking site for the electron donor protein, cytochrome P450 reductase [40,41], whose binding is assisted by complimentary electrostatic interactions: positively charged patches on P450 and acidity on the reductase. By increasing the basicity of the proximal surface, the T409R mutation could promote the redox partner binding and stimulate turnover of CYP3A7.

### 2.7. Concluding Summary

Fetal-specific CYP3A7 regulates the homeostasis of endogenous hormones and differentiation factors as well as detoxifies drugs and other xenobiotics that could reach and harm the fetus during pregnancy. Compared to the closely related and highly promiscuous CYP3A4, the ligand binding and catalytic ability of CYP3A7 is largely diminished. To explain this puzzling phenomenon and obtain the first insight into the protein architecture, we determined the X-ray structure of a surface mutant of CYP3A7, because the wild type protein resists crystallization. The R69G/C77G/K244E/K421A/K422A/K424A mutations do not significantly alter spectral, functional, and molecular properties of CYP3A7, and, thus, the variant can serve as a reliable structural model. Comparative analysis of CYP3A isoforms suggests that a set of structural determinants modulating protein plasticity rather than the active site microenvironment define the ligand binding properties of CYP3A7. In particular, we propose that structural changes in the F-G fragment and the I-E-D-helical core, along with reshaping of the C-terminal loop and H-bonding changes in the substrate channel and β1-sheet, lead to global changes that limit the conformational ensemble, decrease flexibility of the active site, and preclude substrate-dependent spin transitions in the heme, necessary for effective catalysis. Thus, the long-awaited crystal structure of CYP3A7 emphasizes the crucial importance of protein flexibility in substrate binding, expands the knowledge of and helps better understand structure–function relationships of CYP3A enzymes, and warrants further studies to evaluate structural findings and uncover the precise mechanism underlying the reduced ligand binding and functional activity of CYP3A7.

## 3. Materials and Methods

### 3.1. Cloning, Expression, and Purification of CYP3A7

The codon optimized cDNA for the full-length CYP3A7 (UniProt accession number P24462) with the C-terminal 4-histidine affinity tag was synthesized by Synbio Technologies (Monmouth Junction, NJ, USA), cloned into pcWori expression vector, and used as a template for cloning Δ3-22 WT and mutant proteins. Mutations were introduced using QuikChange Site-Directed Mutagenesis Kit (Agilent, Santa Clara, CA, USA) and verified by sequencing. The WT and mutants of CYP3A7 were co-expressed with GroESL in *E. coli* C41 strain. After inoculation into TB media (0.6 L in 2.8 L flasks) supplemented with ampicillin (100 mg/L) and chloramphenicol (34 mg/L), cells were grown at 37 °C and 220 rpm until OD at 600 nm reached 0.6, then temperature was decreased to 30 °C and 5-aminolevulenic acid and isopropyl β-D-1-thiogalactopyranoside (0.5 mM final concentration each) were added to induce protein production. Cells were grown at 100 rpm for 48 h and, after harvesting, resuspended in lysis buffer (0.1 M potassium phosphate pH 7.4, 0.1 M NaCl, 20% glycerol and 5 mM mercaptoethanol) containing 1 mg/L leupeptin and broken by passing through a microfluidizer (Microfluidics, Newton, MA, USA). Cell lysate was incubated with 0.4% Nonidet P-40 (Sigma-Aldrich, St. Louis, MO, USA) for 30 min at 4 °C to solubilize membranes and centrifuged at 32,000× *g* for 60 min to remove cell debris. The supernatant fraction was loaded on HisPur^TM^ Ni-NTA resin (ThermoFisher, Waltham, MA, USA) and washed with 3 volumes of lysis buffer containing 0.2% Nonidet P-40 and 2 volumes of the latter buffer supplemented with 5 mM histidine. Protein was eluted with 0.1 M potassium phosphate pH 7.4, 20% glycerol, 0.2% Nonidet P-40, and 30 mM histidine. The peak fractions were concentrated and loaded on a CM Sepharose Fast Flow column (GE Healthcare, Chicago, Il, USA) equilibrated with 50 mM potassium phosphate pH 7.4, 20% glycerol, and 2 mM DTT. The column was washed overnight with 1 L of CM loading buffer. Protein was eluted with a linear gradient of CM loading buffer vs. 0.125 M potassium phosphate pH 7.4, 0.2 M NaCl, 20% glycerol, and 2 mM DTT. Fractions with A_417/280 nm_ > 1.6 were combined, concentrated, and stored at −80 °C. P450 concentration was determined according to Omura and Sato [42].

### 3.2. Spectral Measurements

Absorbance spectra of CYP3A7 in the absence and presence of various compounds were recorded on Cary 300 spectrophotometer(Agilent, Santa Clara, CA, USA) in 0.1 M potassium phosphate pH 7.4, supplemented with 20% glycerol and 1 mM DTT. Compounds were dissolved in dimethyl sulfoxide (DMSO) to 0.5–5 mM stocks and added to the cuvette in small aliquots to limit the solvent concentration to <1%. Circular dichroism spectra of 3 μM WT and mutant CYP3A7 were recorded on a Jasco J-810 spectropolarimeter (Oklahoma City, Jasco, OK, USA) at room temperature in 50 mM HEPES pH 7.4, using a 1 mm path cuvette.

Equilibrium titrations of CYP3A7 with DCA were conducted on Cary 300 spectrophotometer at ambient temperature in 0.1 M phosphate buffer, pH 7.4, containing 20% glycerol and 1 mM dithiothreitol. DCA was dissolved in DMSO to 0.5–100 mM concentration and added to a protein solution (1–1.5 μM) in small aliquots, with the final solvent concentration <3%. Equal amounts of DMSO were added to the reference cuvette, after which the difference spectra were recorded. K_d_ was derived from hyperbolic fits to titration curves using IgorPro software (WaveMetrics, Inc., Portland, OR, USA).

### 3.3. Activity Assays

The rate of BFC *O*-debenzylation was measured fluorimetrically in a soluble reconstituted system containing 1 μM WT or mutant CYP3A7 with or without 4 μM rat CPR. The reaction was monitored at 37 °C in 0.1 M potassium phosphate pH 7.4, containing catalase and superoxide dismutase (2 Units/mL each) and 0.0025% CHAPS (3-[(3-cholamidopropyl)dimethyl-ammonio]-1-propanesulfonate). After 2 min preincubation with BFC (2–60 μM final concentration), the reaction was initiated with 0.2 mM NADPH or 0.25 mM CuOOH. Formation of 7-hydroxy-4-trifluoro methylcoumarin (HFC) was followed on Hitachi F-7100 fluorimeter (Hitachi, Tokyo, Japan) for 2–3 min (λ_ex_ = 404 nm; λ_em_ = 500 nm), during which the fluorescence increase was linear. Measurements were performed in triplicates. The reaction rates were estimated based on the HFC calibration curve (5–50 nM) obtained under the same experimental conditions.

### 3.4. Gel Filtration

Gel filtration experiments were conducted at 4 °C in 0.1 M potassium phosphate pH 7.4, 0.1 M NaCl, and 1 mM DTT on FPLC Superdex 200 column (0.9 × 30 cm; GE Healthcare) with a 0.15 mL/min flow rate. Protein elution was monitored at 280 nm. Oxidized and NADH-reduced apoptosis inducing factor (58 and 116 kDa, respectively), nitric oxide synthase (320 kDa), and ferritin (440 kDa) were used as molecular standards.

### 3.5. Crystallization and Structure Determination

CYP3A7mut (20 mg/mL in 50 mM phosphate buffer pH 7.4, 10% glycerol, 0.1 M NaCl) was crystallized at ambient temperature by a sitting drop vapor diffusion method vs. 16% PEG 3350, 0.1 M BisTris 7.5, and 0.2 M ammonium sulfate. Crystals were harvested 3 days after setup and cryoprotected with Paratone N before freezing in liquid nitrogen. The X-ray data were collected on the Stanford Synchrotron Radiation Lightsource beamline 9-2 (Menlo Park, CA, USA). Crystal structure was solved by molecular replacement with PHASER [43]. The poly-alanine 5vcc structure of CYP3A4 with residues 207-229 deleted was used as a search model. The initial model was rebuilt with COOT [44] and refined with PHENIX [45]. The N- and C-termini, as well as residues 263-267 and 282-284 were invisible in both molecules of CYP3A7. Simulated annealing omit electron density map for DTT was calculated with PHENIX. Data collection and refinement statistics are summarized in Table 2. The atomic coordinates and structure factors were deposited to the Protein Data Bank with the ID code 7MK8.

## Figures and Tables

**Table 1:** Mutational impact on the BFC O-debenzylase activity of CYP3A7.

CYP3A7	CPR-Supported			CuOOH-Supported		
	V_max_ (min^−1^)	K_m_ (μM)	V_max_/K_m_ (min^−1^ μM^−1^)	V_max_ (min^−1^)	K_m_ (μM)	V_max_/K_m_ (min^−1^ μM^−1^)
WT	0.0036 ± 0.0006	17 ± 3	2.1 × 10^−4^	0.035 ± 0.004	10 ± 2	3.5 × 10^−3^
Mutant	0.0011 ± 0.0003	9 ± 2	1.2 × 10^−4^	0.034 ± 0.005	11 ± 1	3.1 × 10^−3^

**Table 2:** X-ray data collection and structure refinement statistics.

Data statistics		
Space group		C222
Unit cell parameters		*a* = 72 Å, *b* = 205 Å, *c* = 157 Å; α, β, γ = 90°
Molecules per asymmetric unit		2
Resolution range (Å)		85.79–2.15 (2.27–2.15) **^a^**
Total reflections		529,291 (79,567)
Unique reflections		63,247 (9183)
Redundancy		8.4 (8.7)
Completeness		99.5 (99.6)
Average *I*/*σI*		10.7 (1.4)
R_merge_		0.119 (1.628)
R_pim_		0.044 (0.579)
CC ½		0.999 (0.635)
Refinement statistics		
*R_work_*/*R*_free_ **^b^**		20.3/25.3
Number of atoms:		
Protein:	molecule A	3727
	molecule B	3794
Solvent		230
R.m.s. deviations:		
Bond lengths, Å		0.007
Bond angles, °		0.923
Wilson *B*-factor, Å^2^		43
Average *B*-factor, Å^2^:		
Molecule A		59
Molecule B		65
Ligand A		86
Ligand B		100
Solvent		57
Ramachandran plot **^c^** (residues; %)		
Preferred		891 (95.8%)
Allowed		37 (4.0%)
Outliers		2 (0.2%)

**^a^** Values in brackets are for the highest resolution shell; **^b^** *R*_free_ was calculated from a subset of 5% of the data that were excluded during refinement; **^c^** Analyzed with PROCHECK.

## Data Availability

Crystallographic data presented in this study are publicly available in the Protein Data Bank with the ID code 7MK8.

## Supplementary Materials

Supplementary materials can be found at https://www.mdpi.com/article/10.3390/ijms22115831/s1.

## Abbreviations

CYP	Cytochrome P450
CPR	Cytochrome P450 reductase
BFC	7-benzyloxy-4-(trifluoromethyl)coumarin
atRA	All-trans retinoic acid
DHEA	Dihydroepiandrosterone
DHEAS	Dihydroepiandrosterone sulfate
DTT	Dithiothreitol
WT	Wild type
