# Supplementary material for: Structural Basis for the Diminished Ligand Binding and Catalytic Ability of Human Fetal-Specific CYP3A7
Source: Int J Mol Sci. 2021 May 29;22(11):5831. doi: 10.3390/ijms22115831 (PMC8198134; doi:10.3390/ijms22115831)
Supplement: Supplementary file 1 [file ijms-22-05831-s001.zip › PDB_val-report-full_7MK8.pdf]

Preliminary Full wwPDB X-ray Structure Validation Report (i)

Apr 21, 2021 – 06:16 PM EDT

Deposition ID : D_1000256400

This is a Preliminary Full wwPDB X-ray Structure Validation Report.

This report is produced by the wwPDB Deposition System during initial deposition but before annotation of the structure.

We welcome your comments at *validation@mail.wwpdb.org*
A user guide is available at
https://www.wwpdb.org/validation/2017/XrayValidationReportHelp
with specific help available everywhere you see the (i) symbol.

PRELIMINARY VALIDATION REPORT

The following versions of software and data (see references (i)) were used in the production of this report:

MolProbity	:	4.02b-467
Mogul	:	1.8.5 (274361), CSD as541be (2020)
Xtriage (Phenix)	:	1.13
EDS	:	2.18
buster-report	:	1.1.7 (2018)
Percentile statistics	:	20191225.v01 (using entries in the PDB archive December 25th 2019)
Refmac	:	5.8.0158
CCP4	:	7.0.044 (Gargrove)
Ideal geometry (proteins)	:	Engh & Huber (2001)
Ideal geometry (DNA, RNA)	:	Parkinson et al. (1996)
Validation Pipeline (wwPDB-VP)	:	2.18

1 Overall quality at a glance (i)

The following experimental techniques were used to determine the structure:
X-RAY DIFFRACTION

The reported resolution of this entry is 2.15 Å.

Percentile scores (ranging between 0-100) for global validation metrics of the entry are shown in the following graphic. The table shows the number of entries on which the scores are based.

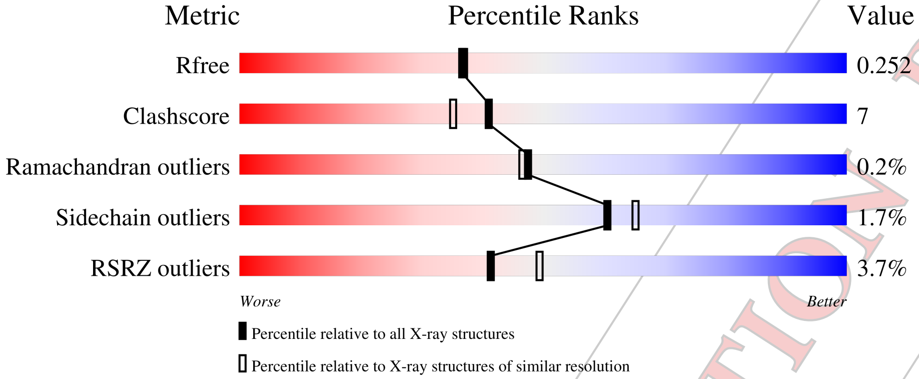

Metric	Whole archive (#Entries)	Similar resolution (#Entries, resolution range(Å))
R_{free}	130704	1479 (2.16-2.16)
Clashscore	141614	1585 (2.16-2.16)
Ramachandran outliers	138981	1560 (2.16-2.16)
Sidechain outliers	138945	1559 (2.16-2.16)
RSRZ outliers	127900	1456 (2.16-2.16)

The table below summarises the geometric issues observed across the polymeric chains and their fit to the electron density. The red, orange, yellow and green segments of the lower bar indicate the fraction of residues that contain outliers for >=3, 2, 1 and 0 types of geometric quality criteria respectively. A grey segment represents the fraction of residues that are not modelled. The numeric value for each fraction is indicated below the corresponding segment, with a dot representing fractions <=5% The upper red bar (where present) indicates the fraction of residues that have poor fit to the electron density. The numeric value is given above the bar.

Mol	Chain	Length	Quality of chain
1	A	519	3% / 75% 13% 11%
2	B	518	3% / 73% 17% 10%

PRELIMINARY VALIDATION REPORT

2 Entry composition (i)

There are 6 unique types of molecules in this entry. The entry contains 7867 atoms, of which 0 are hydrogens and 0 are deuteriums.

In the tables below, the ZeroOcc column contains the number of atoms modelled with zero occupancy, the AltConf column contains the number of residues with at least one atom in alternate conformation and the Trace column contains the number of residues modelled with at most 2 atoms.

- Molecule 1 is a protein called human cytochrome P450 3A7.

Mol	Chain	Residues	Atoms					ZeroOcc	AltConf	Trace
			Total	C	N	O	S			
1	A	462	3727	2433	609	665	20	0	5	0

- Molecule 2 is a protein called human cytochrome P450 3A7.

Mol	Chain	Residues	Atoms					ZeroOcc	AltConf	Trace
			Total	C	N	O	S			
2	B	465	3794	2477	625	672	20	0	10	0

- Molecule 3 is PROTOPORPHYRIN IX CONTAINING FE (three-letter code: HEM) (formula: $C_{34}H_{32}FeN_4O_4$).

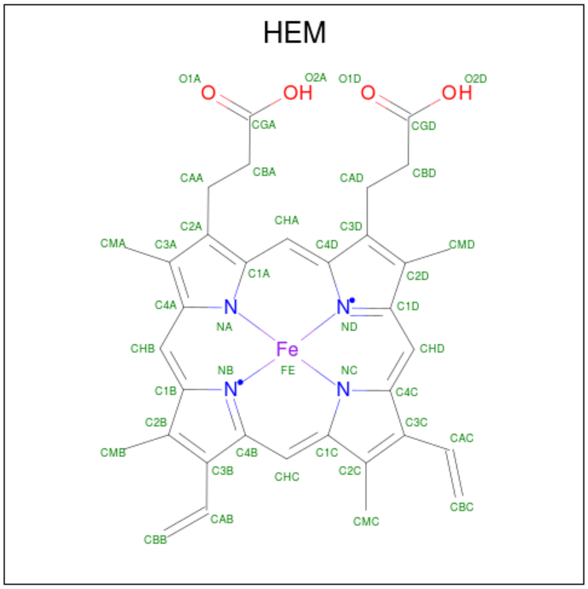

Mol	Chain	Residues	Atoms					ZeroOcc	AltConf
			Total	C	Fe	N	O		
3	A	1	43	34	1	4	4	0	0
3	B	1	43	34	1	4	4	0	0

PRELIMINARY VALIDATION REPORT

- Molecule 4 is SULFATE ION (three-letter code: SO4) (formula: O_4S).

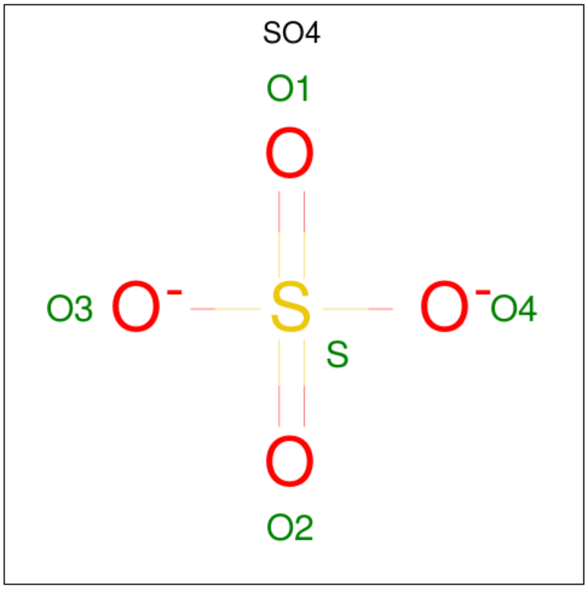

Mol	Chain	Residues	Atoms	ZeroOcc	AltConf
4	A	1	Total O S<br>5 4 1	0	0
4	B	1	Total O S<br>5 4 1	0	0
4	B	1	Total O S<br>5 4 1	0	0

- Molecule 5 is a ligand with the chemical component id DTT but its atom names do not match the existing wwPDB Chemical Component Dictionary definition for DTT. Consequently no firm identification of ligand chemistry can be made. Once the structure is annotated then an identification and diagram will be given here.

Mol	Chain	Residues	Atoms	ZeroOcc	AltConf
5	A	1	Total C O S<br>8 4 2 2	0	0
5	B	1	Total C O S<br>8 4 2 2	0	0

- Molecule 6 is water.

Mol	Chain	Residues	Atoms	ZeroOcc	AltConf
6	S	229	Total O<br>229 229	0	0

PRELIMINARY VALIDATION REPORT

3 Residue-property plots (i)

These plots are drawn for all protein, RNA, DNA and oligosaccharide chains in the entry. The first graphic for a chain summarises the proportions of the various outlier classes displayed in the second graphic. The second graphic shows the sequence view annotated by issues in geometry and electron density. Residues are color-coded according to the number of geometric quality criteria for which they contain at least one outlier: green = 0, yellow = 1, orange = 2 and red = 3 or more. A red dot above a residue indicates a poor fit to the electron density (RSRZ > 2). Stretches of 2 or more consecutive residues without any outlier are shown as a green connector. Residues present in the sample, but not in the model, are shown in grey.

- Molecule 1: human cytochrome P450 3A7

Chain A:

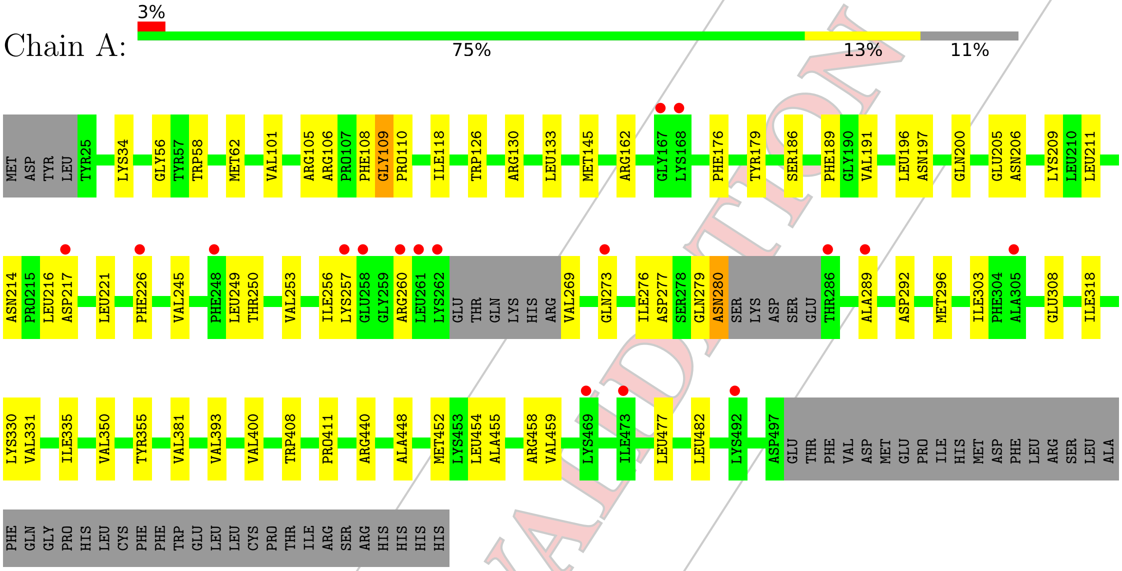

- Molecule 2: human cytochrome P450 3A7

Chain B:

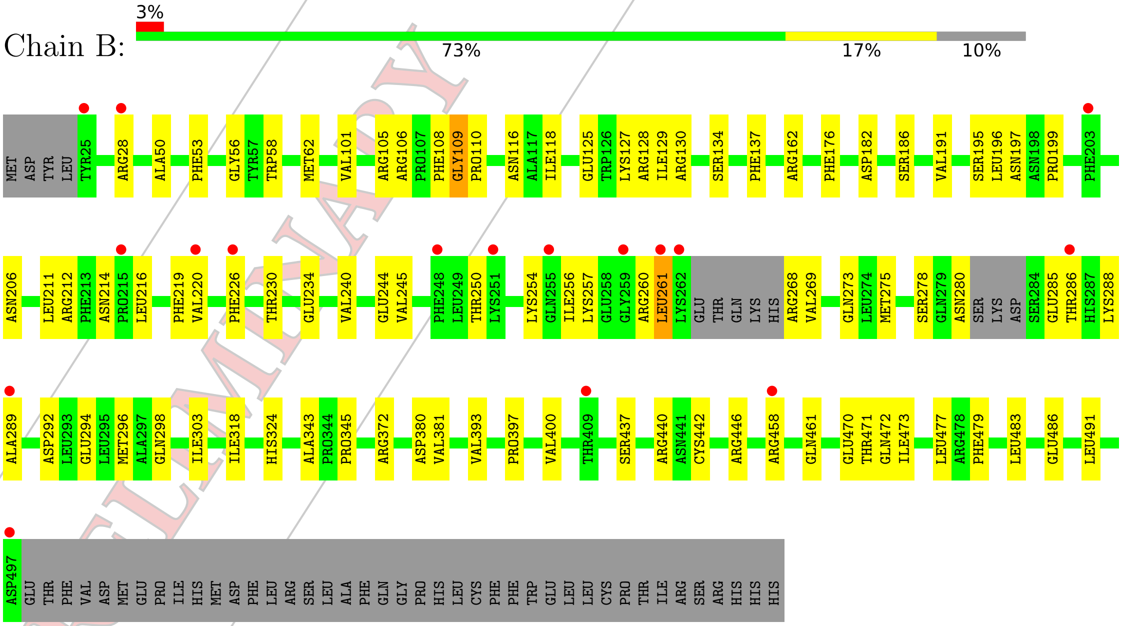

PRELIMINARY VALIDATION REPORT

4 Data and refinement statistics ⓘ

Property	Value	Source
Space group	C 2 2 21	Depositor
Cell constants<br>a, b, c, α, β, γ	72.29Å 205.12Å 156.55Å<br>90.00° 90.00° 90.00°	Depositor
Resolution (Å)	49.67 – 2.15<br>49.67 – 2.15	Depositor<br>EDS
% Data completeness<br>(in resolution range)	99.0 (49.67-2.15)<br>99.0 (49.67-2.15)	Depositor<br>EDS
R_{merge}	(Not available)	Depositor
R_{sym}	(Not available)	Depositor
$< I/\sigma(I) >$ [1]	1.39 (at 2.16Å)	Xtriage
Refinement program	PHENIX 1.11.1_2575	Depositor
R, R_{free}	0.203 , 0.253<br>0.206 , 0.252	Depositor<br>DCC
R_{free} test set	3068 reflections (4.87%)	wwPDB-VP
Wilson B-factor (Å^2)	43.5	Xtriage
Anisotropy	0.598	Xtriage
Bulk solvent k_{sol}(e/Å^3), B_{sol}(Å^2)	0.31 , 47.9	EDS
L-test for twinning[2]	$< \|L\| > = 0.49$, $< L^2 > = 0.32$	Xtriage
Estimated twinning fraction	No twinning to report.	Xtriage
F_o,F_c correlation	0.96	EDS
Total number of atoms	7867	wwPDB-VP
Average B, all atoms (Å^2)	61.0	wwPDB-VP

Xtriage's analysis on translational NCS is as follows: *The largest off-origin peak in the Patterson function is 4.19% of the height of the origin peak. No significant pseudotranslation is detected.*

[1] Intensities estimated from amplitudes.

[2] Theoretical values of $< |L| >$, $< L^2 >$ for acentric reflections are 0.5, 0.333 respectively for untwinned datasets, and 0.375, 0.2 for perfectly twinned datasets.

PRELIMINARY VALIDATION REPORT

5 Model quality (i)

5.1 Standard geometry (i)

Bond lengths and bond angles in the following residue types are not validated in this section: HEM, DTT, SO4

The Z score for a bond length (or angle) is the number of standard deviations the observed value is removed from the expected value. A bond length (or angle) with $|Z| > 5$ is considered an outlier worth inspection. RMSZ is the root-mean-square of all Z scores of the bond lengths (or angles).

Mol	Chain	Bond lengths		Bond angles	
		RMSZ	$\#|Z|>5$	RMSZ	$\#|Z|>5$
1	A	0.38	0/3833	0.56	0/5194
2	B	0.38	0/3917	0.55	0/5305
All	All	0.38	0/7750	0.56	0/10499

There are no bond length outliers.

There are no bond angle outliers.

There are no chirality outliers.

There are no planarity outliers.

5.2 Too-close contacts (i)

In the following table, the Non-H and H(model) columns list the number of non-hydrogen atoms and hydrogen atoms in the chain respectively. The H(added) column lists the number of hydrogen atoms added and optimized by MolProbity. The Clashes column lists the number of clashes within the asymmetric unit, whereas Symm-Clashes lists symmetry-related clashes.

Mol	Chain	Non-H	H(model)	H(added)	Clashes	Symm-Clashes
1	A	3727	0	3836	43	0
2	B	3794	0	3917	64	0
3	A	43	0	30	2	0
3	B	43	0	30	5	0
4	A	5	0	0	0	0
4	B	10	0	0	1	0
5	A	8	0	0	1	0
5	B	8	0	0	0	0
6	S	229	0	0	5	0
All	All	7867	0	7813	110	0

The all-atom clashscore is defined as the number of clashes found per 1000 atoms (including

PRELIMINARY VALIDATION REPORT

hydrogen atoms). The all-atom clashscore for this structure is 7.

All (110) close contacts within the same asymmetric unit are listed below, sorted by their clash magnitude.

Atom-1	Atom-2	Interatomic distance (Å)	Clash overlap (Å)
2:B:285:GLU:HG2	2:B:286:THR:H	1.33	0.91
1:A:280:ASN:HD22	1:A:280:ASN:N	1.77	0.79
1:A:206:ASN:HB3	1:A:249:LEU:HD11	1.64	0.78
2:B:269:VAL:HG12	2:B:269:VAL:O	1.83	0.76
1:A:245:VAL:O	1:A:249:LEU:HD13	1.87	0.75
2:B:127:LYS:HD3	6:S:175:HOH:O	1.89	0.71
1:A:280:ASN:HD22	1:A:280:ASN:H	1.39	0.70
1:A:162:ARG:NH1	1:A:197:ASN:OD1	2.25	0.69
2:B:220:VAL:HG12	2:B:240[A]:VAL:HG21	1.74	0.68
2:B:324:HIS:NE2	4:B:601:SO4:O1	2.25	0.66
1:A:280:ASN:N	1:A:280:ASN:ND2	2.41	0.65
2:B:214:ASN:HD22	2:B:216:LEU:HB2	1.65	0.62
2:B:257:LYS:NZ	2:B:292:ASP:OD2	2.32	0.62
2:B:211:LEU:HD21	2:B:303:ILE:HG22	1.83	0.60
1:A:106:ARG:HD3	1:A:393:VAL:HG21	1.83	0.60
1:A:253:VAL:HA	1:A:256:ILE:HD12	1.85	0.58
2:B:397:PRO:HB2	2:B:400:VAL:HB	1.86	0.57
1:A:118:ILE:HD11	1:A:130:ARG:HD2	1.87	0.57
2:B:212:ARG:NH2	6:S:208:HOH:O	2.35	0.57
1:A:179:TYR:CZ	1:A:455:ALA:HB2	2.41	0.56
1:A:257:LYS:NZ	1:A:292:ASP:OD2	2.38	0.56
2:B:260:ARG:HH11	2:B:273:GLN:HG2	1.71	0.55
2:B:109:GLY:H	2:B:110:PRO:HD2	1.72	0.54
1:A:109:GLY:H	1:A:110:PRO:HD2	1.73	0.54
2:B:458[A]:ARG:NH2	2:B:461:GLN:OE1	2.35	0.54
1:A:126:TRP:CZ2	1:A:440:ARG:HD2	2.42	0.54
1:A:58:TRP:CZ2	1:A:477:LEU:HB2	2.42	0.54
2:B:176:PHE:CE2	2:B:318:ILE:HG13	2.42	0.54
2:B:269:VAL:O	2:B:269:VAL:CG1	2.52	0.54
1:A:250:THR:HG22	1:A:296:MET:HG3	1.89	0.54
2:B:260:ARG:NH1	2:B:273:GLN:HG2	2.23	0.54
2:B:162:ARG:NH1	2:B:197:ASN:OD1	2.41	0.54
2:B:110:PRO:HG2	2:B:240[B]:VAL:HG13	1.90	0.53
2:B:105[B]:ARG:NH2	3:B:500:HEM:O2D	2.34	0.53
2:B:62[B]:MET:HG2	2:B:400:VAL:HG22	1.89	0.53
1:A:108:PHE:H	1:A:109:GLY:HA2	1.73	0.53
2:B:108:PHE:H	2:B:109:GLY:HA2	1.73	0.53
1:A:308:GLU:HG2	1:A:482:LEU:HD12	1.90	0.53

Continued on next page...

PRELIMINARY VALIDATION REPORT

Continued from previous page...

Atom-1	Atom-2	Interatomic distance (Å)	Clash overlap (Å)
2:B:470:GLU:N	2:B:470:GLU:OE1	2.42	0.52
1:A:216:LEU:HD23	1:A:221:LEU:HD21	1.92	0.52
2:B:458[B]:ARG:NH1	6:S:203:HOH:O	2.41	0.52
1:A:108:PHE:N	1:A:109:GLY:HA2	2.25	0.51
2:B:108:PHE:N	2:B:109:GLY:HA2	2.25	0.51
3:B:500:HEM:HBB2	3:B:500:HEM:HMB2	1.94	0.50
1:A:189:PHE:CZ	1:A:249:LEU:HD23	2.47	0.49
2:B:380:ASP:O	2:B:381:VAL:HG13	2.12	0.49
1:A:205:GLU:O	1:A:209:LYS:HG3	2.13	0.49
2:B:442:CYS:HB2	3:B:500:HEM:NA	2.27	0.49
1:A:62[B]:MET:HG2	1:A:400:VAL:HG22	1.95	0.48
2:B:285:GLU:HG2	2:B:286:THR:N	2.14	0.48
1:A:350:VAL:HG21	1:A:454:LEU:HD23	1.96	0.48
2:B:182:ASP:OD2	2:B:195[A]:SER:OG	2.20	0.48
1:A:408:TRP:HB2	1:A:411:PRO:HB3	1.95	0.48
2:B:137:PHE:O	2:B:446:ARG:NH2	2.30	0.47
2:B:230:THR:O	2:B:234:GLU:HG3	2.14	0.47
1:A:277:ASP:O	1:A:280:ASN:ND2	2.48	0.46
2:B:58[B]:TRP:CZ2	2:B:477:LEU:HB2	2.51	0.46
2:B:471:THR:HG22	2:B:473:ILE:HG13	1.98	0.46
2:B:250:THR:HG22	2:B:296:MET:HG3	1.96	0.46
5:A:700:DTT:S05	6:S:23:HOH:O	2.61	0.46
2:B:254:LYS:HA	2:B:254:LYS:HD2	1.80	0.45
2:B:380:ASP:O	2:B:381:VAL:CG1	2.65	0.45
2:B:50:ALA:HA	2:B:53:PHE:CE2	2.52	0.45
2:B:343:ALA:HB2	6:S:212:HOH:O	2.16	0.45
2:B:106:ARG:HD3	2:B:393:VAL:HG21	2.00	0.44
1:A:331:VAL:O	1:A:335:ILE:HG13	2.16	0.44
1:A:56:GLY:HA3	1:A:58:TRP:CZ3	2.53	0.44
1:A:176:PHE:CE2	1:A:318:ILE:HG13	2.53	0.44
2:B:278:SER:C	2:B:280:ASN:H	2.21	0.44
2:B:275:MET:O	2:B:278:SER:OG	2.33	0.44
1:A:126:TRP:CE2	1:A:440:ARG:HD2	2.52	0.44
2:B:118:ILE:HD11	2:B:130:ARG:HD2	1.99	0.44
1:A:257:LYS:HG2	1:A:276:ILE:HD11	1.99	0.43
2:B:186:SER:HA	2:B:191:VAL:O	2.18	0.43
2:B:442:CYS:HB2	3:B:500:HEM:C1A	2.53	0.43
1:A:279:GLN:HA	1:A:289:ALA:HB1	2.00	0.43
1:A:133:LEU:HD23	1:A:133:LEU:HA	1.87	0.43
2:B:58[A]:TRP:CZ2	2:B:477:LEU:HB2	2.53	0.43
2:B:294:GLU:O	2:B:298:GLN:HG2	2.18	0.43

Continued on next page...

PRELIMINARY VALIDATION REPORT

Continued from previous page...

Atom-1	Atom-2	Interatomic distance (Å)	Clash overlap (Å)
1:A:217:ASP:OD1	1:A:217:ASP:N	2.52	0.43
2:B:28:ARG:HD3	2:B:28:ARG:HA	1.83	0.43
1:A:448:ALA:O	1:A:452:MET:HG3	2.19	0.42
2:B:196:LEU:HA	2:B:196:LEU:HD23	1.80	0.42
2:B:472:GLN:NE2	2:B:486:GLU:OE2	2.49	0.42
1:A:186:SER:HA	1:A:191:VAL:O	2.18	0.42
2:B:191:VAL:HG13	2:B:256:ILE:HD13	2.01	0.42
2:B:458[A]:ARG:HD3	2:B:458[A]:ARG:HA	1.80	0.42
1:A:330:LYS:HD3	1:A:355:TYR:CZ	2.55	0.42
2:B:105[B]:ARG:HH21	3:B:500:HEM:CGD	2.28	0.42
1:A:196:LEU:HD23	1:A:196:LEU:HA	1.81	0.42
2:B:244:GLU:HG3	2:B:245:VAL:HG23	2.02	0.42
2:B:125:GLU:N	2:B:125:GLU:OE1	2.53	0.42
2:B:288:LYS:HG2	2:B:289:ALA:N	2.35	0.42
1:A:145:MET:HG3	1:A:269:VAL:O	2.19	0.42
1:A:211:LEU:HD21	1:A:303:ILE:HG22	2.01	0.42
2:B:56:GLY:HA2	2:B:479:PHE:HD1	1.85	0.42
2:B:101:VAL:HG21	2:B:381:VAL:HG11	2.01	0.42
2:B:116:ASN:O	2:B:129:ILE:HD13	2.20	0.42
1:A:101:VAL:HG21	1:A:381:VAL:HG11	2.01	0.41
3:A:500:HEM:HMC2	3:A:500:HEM:HBC2	2.02	0.41
1:A:214:ASN:HD22	1:A:216:LEU:HB2	1.85	0.41
2:B:128:ARG:NH2	2:B:289:ALA:O	2.54	0.41
2:B:345:PRO:HD2	2:B:458[B]:ARG:NH1	2.36	0.41
1:A:260:ARG:HD3	1:A:273:GLN:OE1	2.20	0.41
2:B:261:LEU:HD12	2:B:261:LEU:HA	1.80	0.41
2:B:471:THR:OG1	2:B:491:LEU:HD23	2.21	0.41
1:A:105:ARG:HH21	3:A:500:HEM:CGD	2.33	0.40
2:B:206:ASN:O	2:B:245:VAL:HG11	2.22	0.40
2:B:477:LEU:HD13	2:B:483:LEU:HD11	2.03	0.40
2:B:196:LEU:O	2:B:199:PRO:HD3	2.21	0.40

There are no symmetry-related clashes.

5.3 Torsion angles (i)

5.3.1 Protein backbone (i)

In the following table, the Percentiles column shows the percent Ramachandran outliers of the chain as a percentile score with respect to all X-ray entries followed by that with respect to entries of similar resolution.

PRELIMINARY VALIDATION REPORT

The Analysed column shows the number of residues for which the backbone conformation was analysed, and the total number of residues.

Mol	Chain	Analysed	Favoured	Allowed	Outliers	Percentiles	
1	A	461/519 (89%)	443 (96%)	17 (4%)	1 (0%)	47	46
2	B	469/518 (90%)	448 (96%)	20 (4%)	1 (0%)	47	46
All	All	930/1037 (90%)	891 (96%)	37 (4%)	2 (0%)	47	46

All (2) Ramachandran outliers are listed below:

Mol	Chain	Res	Type
1	A	109	GLY
2	B	109	GLY

5.3.2 Protein sidechains (i)

In the following table, the Percentiles column shows the percent sidechain outliers of the chain as a percentile score with respect to all X-ray entries followed by that with respect to entries of similar resolution.

The Analysed column shows the number of residues for which the sidechain conformation was analysed, and the total number of residues.

Mol	Chain	Analysed	Rotameric	Outliers	Percentiles	
1	A	422/472 (89%)	416 (99%)	6 (1%)	67	72
2	B	430/471 (91%)	422 (98%)	8 (2%)	57	61
All	All	852/943 (90%)	838 (98%)	14 (2%)	60	67

All (14) residues with a non-rotameric sidechain are listed below:

Mol	Chain	Res	Type
1	A	34	LYS
1	A	200	GLN
1	A	226	PHE
1	A	280	ASN
1	A	458	ARG
1	A	459	VAL
2	B	134	SER
2	B	219	PHE
2	B	226	PHE
2	B	261	LEU
2	B	268	ARG

Continued on next page...

PRELIMINARY VALIDATION REPORT

Continued from previous page...

Mol	Chain	Res	Type
2	B	372	ARG
2	B	437	SER
2	B	440	ARG

Sometimes sidechains can be flipped to improve hydrogen bonding and reduce clashes. All (4) such sidechains are listed below:

Mol	Chain	Res	Type
1	A	280	ASN
2	B	214	ASN
2	B	423	ASN
2	B	426	ASN

5.3.3 RNA (i)

There are no RNA molecules in this entry.

5.4 Non-standard residues in protein, DNA, RNA chains (i)

There are no non-standard protein/DNA/RNA residues in this entry.

5.5 Carbohydrates (i)

There are no monosaccharides in this entry.

5.6 Ligand geometry (i)

Of 7 ligands modelled in this entry, 2 could not be matched to an existing wwPDB Chemical Component Dictionary definition at this stage - leaving 5 for Mogul analysis.

In the following table, the Counts columns list the number of bonds (or angles) for which Mogul statistics could be retrieved, the number of bonds (or angles) that are observed in the model and the number of bonds (or angles) that are defined in the Chemical Component Dictionary. The Link column lists molecule types, if any, to which the group is linked. The Z score for a bond length (or angle) is the number of standard deviations the observed value is removed from the expected value. A bond length (or angle) with $|Z| > 2$ is considered an outlier worth inspection. RMSZ is the root-mean-square of all Z scores of the bond lengths (or angles).

Mol	Type	Chain	Res	Link	Bond lengths			Bond angles		
					Counts	RMSZ	$\#\|Z\| > 2$	Counts	RMSZ	$\#\|Z\| > 2$
4	SO4	A	602	-	4,4,4	0.14	0	6,6,6	0.05	0

PRELIMINARY VALIDATION REPORT

Mol	Type	Chain	Res	Link	Bond lengths			Bond angles		
					Counts	RMSZ	$\#\|Z\|>2$	Counts	RMSZ	$\#\|Z\|>2$
3	HEM	B	500	6,2	27,50,50	1.87	6 (22%)	17,82,82	1.79	5 (29%)
4	SO4	B	600	-	4,4,4	0.16	0	6,6,6	0.32	0
3	HEM	A	500	6,1	27,50,50	1.91	5 (18%)	17,82,82	1.64	5 (29%)
4	SO4	B	601	-	4,4,4	0.12	0	6,6,6	0.17	0

In the following table, the Chirals column lists the number of chiral outliers, the number of chiral centers analysed, the number of these observed in the model and the number defined in the Chemical Component Dictionary. Similar counts are reported in the Torsion and Rings columns. '-' means no outliers of that kind were identified.

Mol	Type	Chain	Res	Link	Chirals	Torsions	Rings
3	HEM	A	500	6,1	-	0/6/54/54	-
3	HEM	B	500	6,2	-	0/6/54/54	-

All (11) bond length outliers are listed below:

Mol	Chain	Res	Type	Atoms	Z	Observed(Å)	Ideal(Å)
3	A	500	HEM	C3B-CAB	4.01	1.56	1.47
3	B	500	HEM	C3B-CAB	3.89	1.55	1.47
3	A	500	HEM	C3C-CAC	3.85	1.55	1.47
3	B	500	HEM	C3C-C2C	-3.82	1.35	1.40
3	B	500	HEM	C3C-CAC	3.78	1.55	1.47
3	A	500	HEM	C3C-C2C	-3.61	1.35	1.40
3	B	500	HEM	C3B-C2B	-3.57	1.35	1.40
3	A	500	HEM	C3B-C2B	-3.43	1.35	1.40
3	B	500	HEM	CAA-C2A	2.67	1.56	1.52
3	A	500	HEM	CAA-C2A	2.66	1.56	1.52
3	B	500	HEM	CAD-C3D	2.02	1.55	1.52

All (10) bond angle outliers are listed below:

Mol	Chain	Res	Type	Atoms	Z	Observed(^{o})	Ideal(^{o})
3	B	500	HEM	CMA-C3A-C4A	-3.14	123.64	128.46
3	A	500	HEM	CMD-C2D-C1D	-2.99	123.87	128.46
3	A	500	HEM	CBD-CAD-C3D	-2.72	107.46	112.48
3	B	500	HEM	CMB-C2B-C3B	2.56	129.48	124.68
3	B	500	HEM	C1D-C2D-C3D	2.50	108.73	107.00
3	B	500	HEM	CMD-C2D-C1D	-2.43	124.73	128.46
3	A	500	HEM	CMC-C2C-C3C	2.14	128.68	124.68
3	B	500	HEM	CMC-C2C-C3C	2.12	128.65	124.68
3	A	500	HEM	CBA-CAA-C2A	-2.11	108.60	112.49
3	A	500	HEM	CMA-C3A-C4A	-2.06	125.30	128.46

PRELIMINARY VALIDATION REPORT

There are no chirality outliers.

There are no torsion outliers.

There are no ring outliers.

3 monomers are involved in 8 short contacts:

Mol	Chain	Res	Type	Clashes	Symm-Clashes
3	B	500	HEM	5	0
3	A	500	HEM	2	0
4	B	601	SO4	1	0

The following is a two-dimensional graphical depiction of Mogul quality analysis of bond lengths, bond angles, torsion angles, and ring geometry for all instances of the Ligand of Interest. In addition, ligands with molecular weight > 250 and outliers as shown on the validation Tables will also be included. For torsion angles, if less then 5% of the Mogul distribution of torsion angles is within 10 degrees of the torsion angle in question, then that torsion angle is considered an outlier. Any bond that is central to one or more torsion angles identified as an outlier by Mogul will be highlighted in the graph. For rings, the root-mean-square deviation (RMSD) between the ring in question and similar rings identified by Mogul is calculated over all ring torsion angles. If the average RMSD is greater than 60 degrees and the minimal RMSD between the ring in question and any Mogul-identified rings is also greater than 60 degrees, then that ring is considered an outlier. The outliers are highlighted in purple. The color gray indicates Mogul did not find sufficient equivalents in the CSD to analyse the geometry.

PRELIMINARY VALIDATION REPORT

Ligand HEM B 500

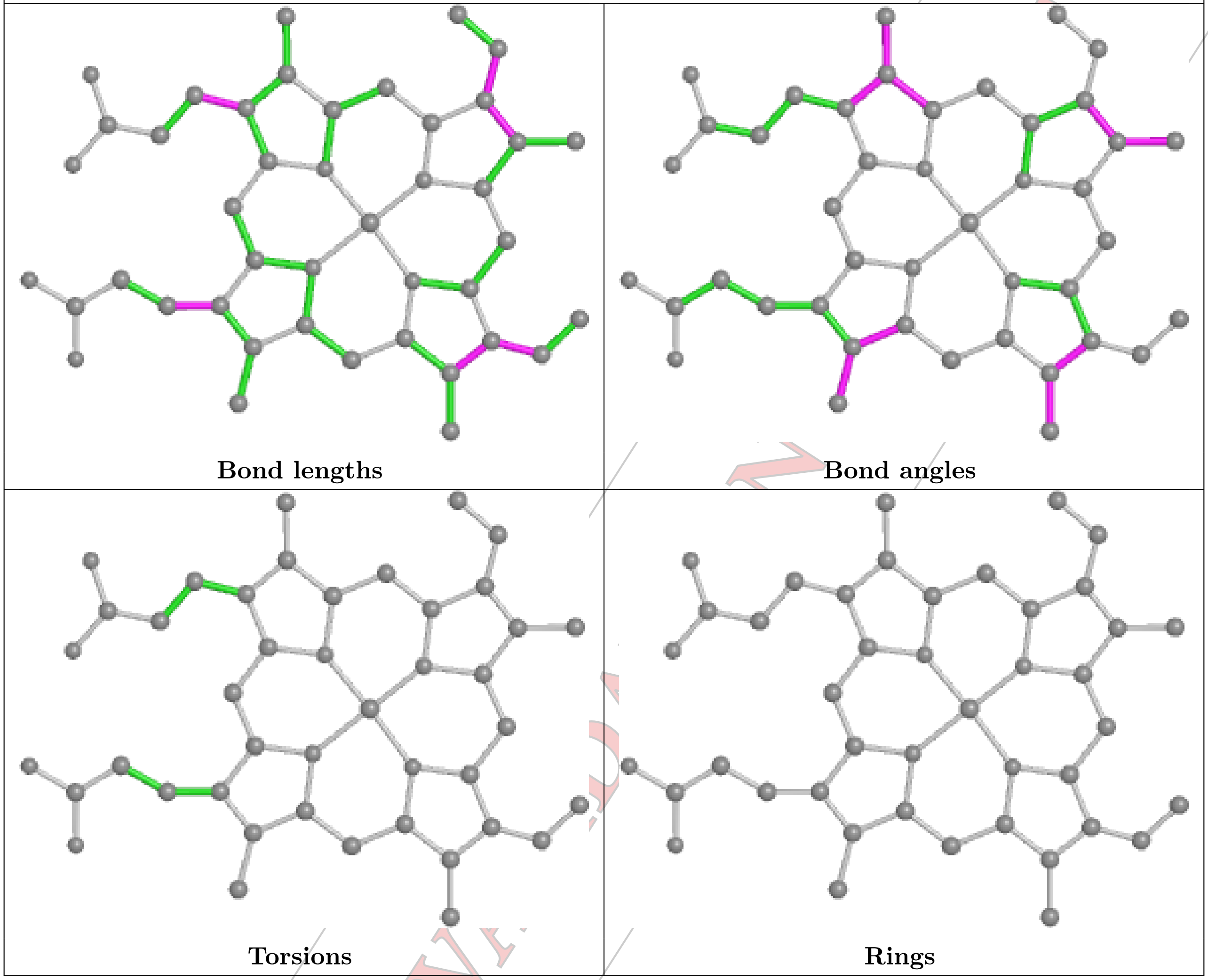

PRELIMINARY VALIDATION

Ligand HEM A 500

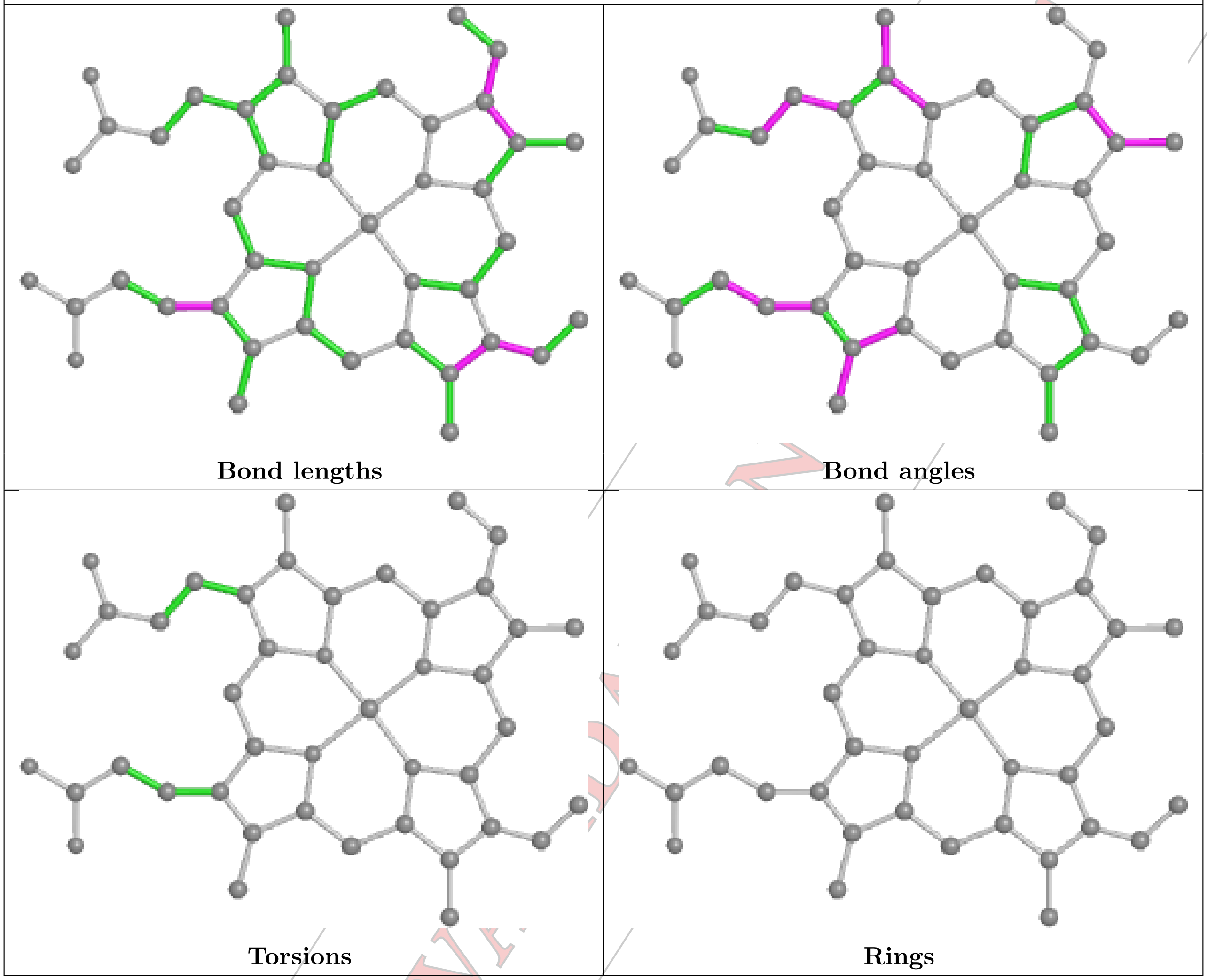

5.7 Other polymers (i)

There are no such residues in this entry.

5.8 Polymer linkage issues (i)

There are no chain breaks in this entry.

6 Fit of model and data (i)

6.1 Protein, DNA and RNA chains (i)

In the following table, the column labelled '#RSRZ> 2' contains the number (and percentage) of RSRZ outliers, followed by percent RSRZ outliers for the chain as percentile scores relative to all X-ray entries and entries of similar resolution. The OWAB column contains the minimum, median, 95th percentile and maximum values of the occupancy-weighted average B-factor per residue. The column labelled 'Q< 0.9' lists the number of (and percentage) of residues with an average occupancy less than 0.9.

Mol	Chain	Analysed	<RSRZ>	#RSRZ>2			OWAB(Å^2)	Q<0.9
1	A	462/519 (89%)	0.14	17 (3%)	41	49	33, 54, 94, 136	0
2	B	465/518 (89%)	0.11	17 (3%)	41	49	39, 60, 99, 141	0
All	All	927/1037 (89%)	0.12	34 (3%)	41	49	33, 57, 96, 141	0

All (34) RSRZ outliers are listed below:

Mol	Chain	Res	Type	RSRZ
2	B	248	PHE	4.7
2	B	28	ARG	4.7
1	A	261	LEU	4.2
2	B	220	VAL	3.9
2	B	259	GLY	3.8
2	B	261	LEU	3.3
2	B	226	PHE	3.2
2	B	262	LYS	3.2
1	A	473	ILE	3.1
1	A	286	THR	3.0
1	A	248	PHE	3.0
2	B	25	TYR	3.0
1	A	273	GLN	3.0
1	A	226	PHE	2.9
2	B	251	LYS	2.9
2	B	289	ALA	2.8
2	B	203	PHE	2.8
2	B	497	ASP	2.7
2	B	409	THR	2.7
1	A	262	LYS	2.5
2	B	286	THR	2.5
1	A	168	LYS	2.5
1	A	305	ALA	2.4
1	A	257	LYS	2.4

Continued on next page...

PRELIMINARY VALIDATION REPORT

Continued from previous page...

Mol	Chain	Res	Type	RSRZ
1	A	289	ALA	2.2
2	B	215	PRO	2.2
2	B	255	GLN	2.2
1	A	469	LYS	2.1
2	B	458[A]	ARG	2.1
1	A	258	GLU	2.1
1	A	492	LYS	2.1
1	A	260	ARG	2.1
1	A	217	ASP	2.0
1	A	167	GLY	2.0

6.2 Non-standard residues in protein, DNA, RNA chains (i)

There are no non-standard protein/DNA/RNA residues in this entry.

6.3 Carbohydrates (i)

There are no monosaccharides in this entry.

6.4 Ligands (i)

In the following table, the Atoms column lists the number of modelled atoms in the group and the number defined in the chemical component dictionary. The B-factors column lists the minimum, median, 95th percentile and maximum values of B factors of atoms in the group. The column labelled 'Q< 0.9' lists the number of atoms with occupancy less than 0.9.

Mol	Type	Chain	Res	Atoms	RSCC	RSR	B-factors(Å^2)	Q<0.9
5	DTT	A	700	8/?	0.81	0.20	70,85,91,95	0
4	SO4	B	601	5/?	0.82	0.18	129,134,137,143	0
5	DTT	B	700	8/?	0.86	0.14	89,100,103,106	0
4	SO4	A	602	5/?	0.92	0.19	129,130,134,137	0
3	HEM	A	500	43/?	0.98	0.14	28,38,46,50	0
3	HEM	B	500	43/?	0.98	0.15	32,41,48,50	0
4	SO4	B	600	5/?	0.99	0.09	45,58,65,67	0

The following is a graphical depiction of the model fit to experimental electron density of all instances of the Ligand of Interest. In addition, ligands with molecular weight > 250 and outliers as shown on the geometry validation Tables will also be included. Each fit is shown from different orientation to approximate a three-dimensional view.

PRELIMINARY VALIDATION REPORT

Electron density around HEM A 500:

$2mF_o$-DF_c (at 0.7 rmsd) in gray
mF_o-DF_c (at 3 rmsd) in purple (negative)
and green (positive)

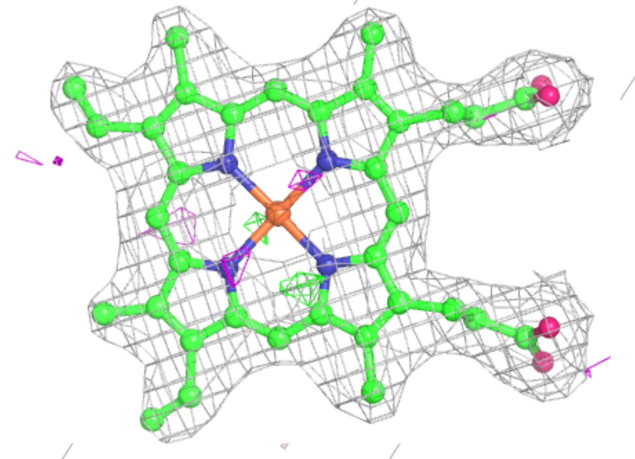

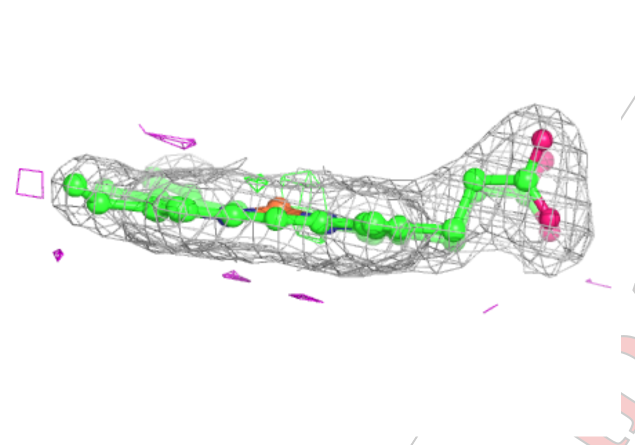

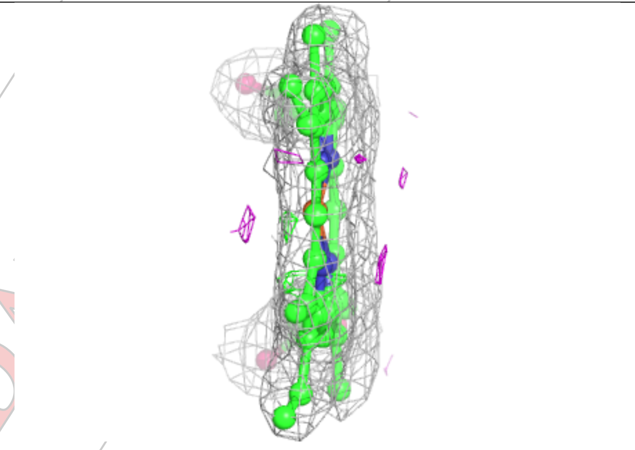

PRELIMINARY VALI

Electron density around HEM B 500:

$2mF_o$-DF_c (at 0.7 rmsd) in gray
mF_o-DF_c (at 3 rmsd) in purple (negative)
and green (positive)

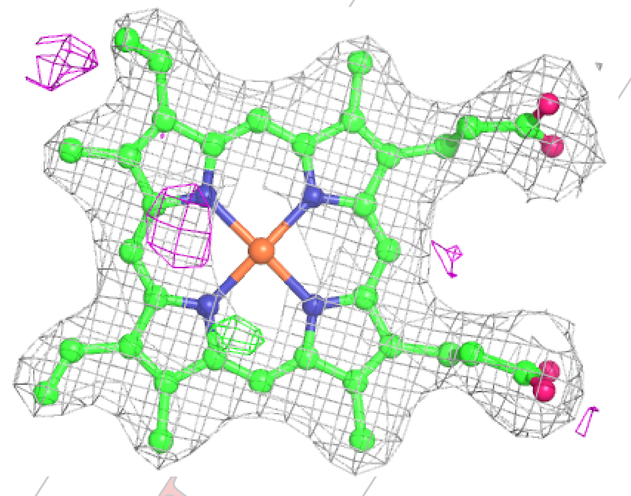

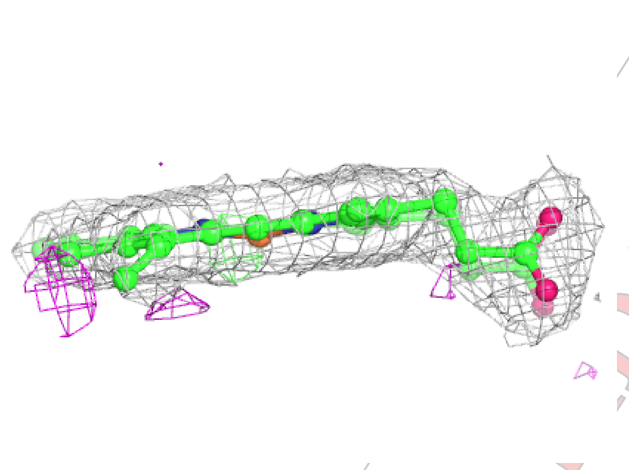

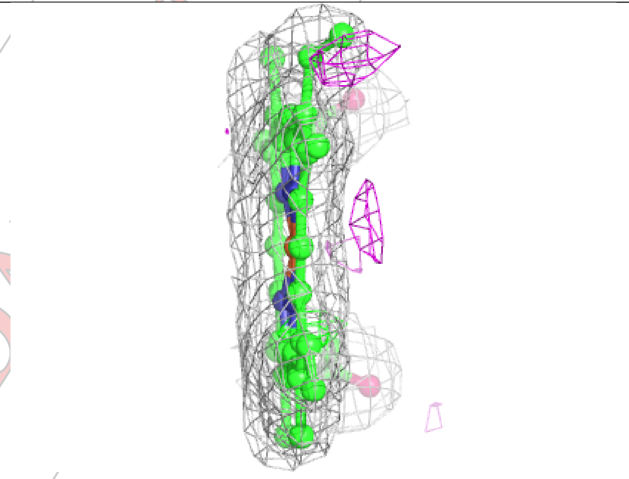

6.5 Other polymers ⓘ

There are no such residues in this entry.

PRELIMINARY VALIDATION
