# Supplementary material for: Structural Basis for the Diminished Ligand Binding and Catalytic Ability of Human Fetal-Specific CYP3A7
Source: Int J Mol Sci. 2021 May 29;22(11):5831. doi: 10.3390/ijms22115831 (PMC8198134; doi:10.3390/ijms22115831)
Supplement: Supplementary file 1 [file ijms-22-05831-s001.zip › Supplementary Figure.pdf]

Supplementary Material

Structural Basis for the Diminished Ligand Binding and Catalytic Ability of Human Fetal-Specific CYP3A7

Irina F. Sevrioukova

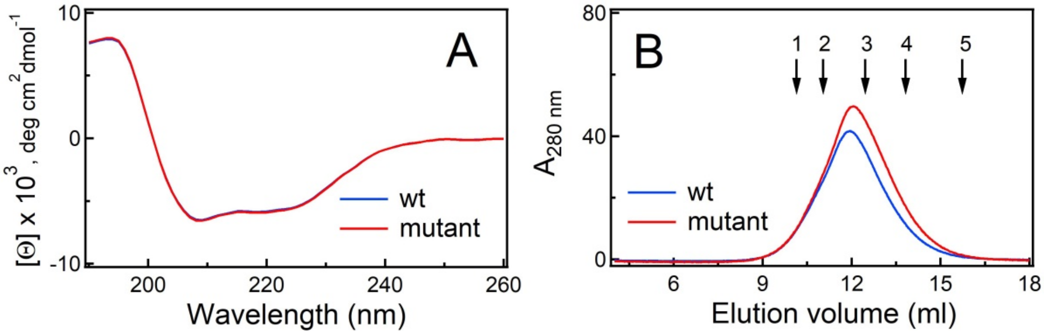

Figure S1. Circular dichroism spectra (**A**) and gel filtration elution profiles (**B**) of WT and R69G/C77G/K244E/K421A/K422A/K424A CYP3A7. CD spectra of 3 μM proteins were recorded in 50 mM HEPES pH 7.4 at room temperature. Gel filtration experiments were conducted at 4 °C in 0.1 M potassium phosphate buffer pH 7.4, containing 0.1 M NaCl, 10% glycerol and 1 mM DTT. Both WT and mutant CYP3A4 were heterogenic and eluted as a mixture of dimers and higher molecular weight oligomers. Molecular standards: 1—ferritin (440 kDa); 2—nitric oxide synthase (320 kDa); 3 and 4—NADH-reduced dimer (116 kDa) and oxidized monomer (58 kDa) of apoptosis inducing factor, respectively; 5—cytochrome *c* (12 kDa). Molecular weight of Δ3-22 CYP3A7 is 56 kDa.

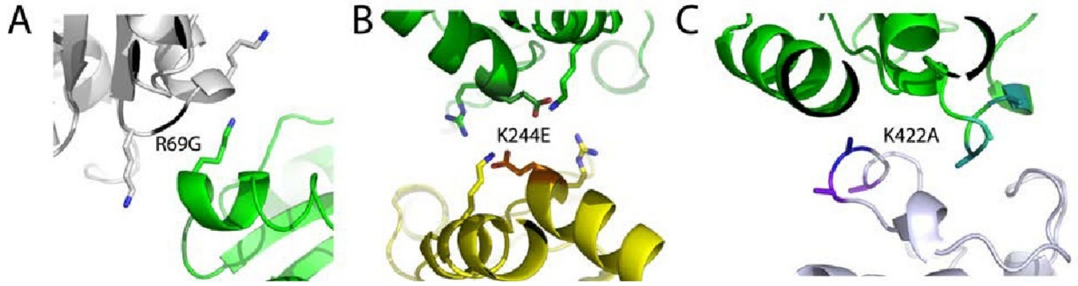

Figure S2. (**A**–**C**) R69G, K244E and K422A residues, respectively, are positioned at the crystal packing interface and could help crystal formation by allowing closer intermolecular contacts.

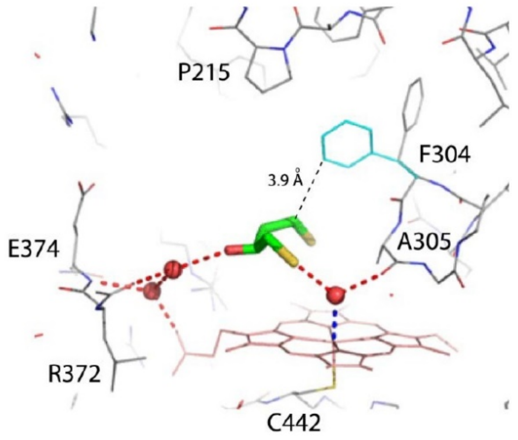

Figure S3. Rotameric change in F304 from 'upward' to 'inward' conformation (in cyan) does not lead to steric clashing with DTT (shown in green sticks). Red spheres are water molecules bridging DTT to the heme and nearby residues.

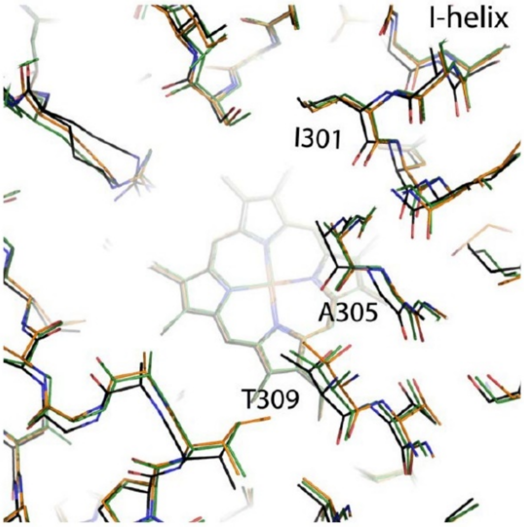

Figure S4. Relative positioning of the I-helix in CYP3A enzymes. CYP3A4 (5vcc), CYP3A5 (6mjm) and CYP3A7 (molecule A) are rendered in orange, green and black, respectively. Relative to CYP3A4, residues I301, A305 and T309 in CYP3A7 are shifted toward the heme by 0.43, 0.73 and 0.97 Å, respectively.

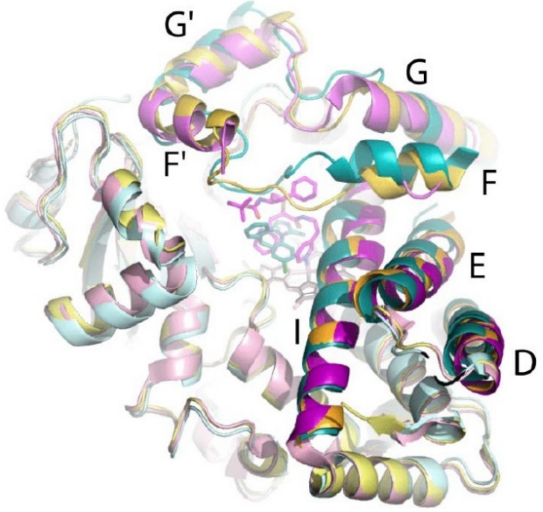

Figure S5. Superposition of ligand-free (5vcc; yellow/orange), midazolam- (5te8; cyan/teal) and **4c**-bound (6dab; pink/magenta) CYP3A4 shows notable ligand-dependent reorganization in the F-G fragment and the I-E-D-helical core.
